# Vaginal microbiota: different roles of lactobacilli and community instability in chronic vulvovaginal discomfort

**DOI:** 10.3389/fcimb.2025.1636873

**Published:** 2025-08-18

**Authors:** Vladimír Buchta, Jana Nekvindová, Daniel Leško, Filip Vrbacký, Peter Veščičík, Zuzana Uhlířová, Ctirad Andrýs, Radka Bolehovská, Marian Kacerovský, Jiří Špaček, Alena Mrkvicová, Hana Skalská, Lenka Plíšková

**Affiliations:** ^1^ Department of Clinical Microbiology, University Hospital Hradec Kralove, Hradec Kralove, Czechia; ^2^ Charles University, Faculty of Medicine in Hradec Kralove, Hradec Kralove, Czechia; ^3^ Department of Clinical Biochemistry and Diagnostics, University Hospital Hradec Kralove, Hradec Kralove, Czechia; ^4^ Department of Obstetrics and Gynecology, University Hospital Hradec Kralove, Hradec Kralove, Czechia; ^5^ 4th Department of Internal Medicine – Hematology, University Hospital Hradec Kralove, Hradec Kralove, Czechia; ^6^ Department of Clinical Immunology and Allergology, University Hospital Hradec Kralove, Hradec Kralove, Czechia; ^7^ Department of Informatics and Quantitative Methods, Faculty of Informatics and Management, University of Hradec Kralove, Hradec Kralove, Czechia

**Keywords:** chronic vulvovaginal discomfort, vaginal microbiota, community state type (CST), CST shift, CST stability, *Lactobacillus iners*, next generation sequencing (NGS)

## Abstract

**Background:**

Chronic vulvovaginal discomfort (CVD) is a complex syndrome with many unresolved questions regarding its etiology, diagnosis, and management in relation to the vaginal microbiota.

**Methods:**

Cervicovaginal fluid of 91 CVD patients and 35 healthy controls was obtained at the beginning and end of the follow-up period. The bacterial community state types (CST) in the vagina were assessed using next-generation sequencing (NGS). CVD patients were divided into four study groups by etiology: non-specific, yeast, bacterial, and mixed.

**Results:**

The vaginal microbiota was characterized by 1) predominance of CST3 in all study groups, 2) a relatively higher proportion of CST2 (29.2%) and CST4 (20.0%) in the non-specific group and controls, respectively, 3) lack of CST4 (4.0%) in the yeast group, and 4) an effect of clinical status (CVD vs. health) on CST stability and microbiota composition. The vaginal environment was shaped by lactobacilli except for CST4. CVD patients had a higher proportion of G-positive cocci than controls; the non-specific group had significantly higher *L. gasseri* abundance than other CVD etiologies. There was a negative correlation between *L. crispatus* and *L. iners*, between G-positive cocci and *L. iners*, and a positive correlation between G-positive cocci and non-*bivia Prevotella* species. CST3 in CVD patients represented the most stable CST and was the community to which other CSTs were most often converted, whereas in healthy controls, CST3 was the most labile CST, with a preferential shift to CST4. The distribution of unstable CSTs was similar in both main cohorts, but within CVD group, non-specific etiology showed significantly higher proportion of unstable CSTs and *L. gasseri*.

**Conclusion:**

Our results revealed an opposing trend in the abundance of *L. iners* and *L. gasseri* between CVD patients and healthy controls, depending on CST stability. We hypothesize that the increased prevalence of CST2 and CST3 may result either from persistent CVD-associated pressure (CST2 and partially CST3), or from enhanced community stability (CST3). The finding that the importance and behavior of *Lactobacillus* species depend on the patient’s clinical status and microbiota context (CST) should contribute to more accurate diagnoses (correct interpretation of laboratory findings) and management of CVD.

## 1 Introduction

Vulvovaginal discomfort of bacterial and yeast origin is one of the most common reasons for gynecological visits (Nyirjesy et al., 2006; Fischer and Bradford, 2011; van de Wijgert and Jespers, 2017). Some women develop a chronic form of this syndrome (CVD), which is difficult to treat, often because of an incorrect diagnostic approach at the first visit and inadequate treatment options, which can beget long-lasting problems and frustration for the patient (Bradshaw et al., 2006; Nyirjesy, 2014). The study of vaginal microbiota has undergone turbulent development over the last two decades and has provided several new insights (Ma et al., 2012; van de Wijgert et al., 2014; Lev-Sagie et al., 2022; Ventolini et al., 2022; Verstraelen et al., 2022; Lee et al., 2023). With the introduction of new molecular techniques such as next generation sequencing, the image of a uniform microbiota consisting of one species, *Lactobacillus acidophilus* was replaced by the concept of a dynamic vaginal microbiome based on the existence of several bacterial community state types (CSTs), with a different *Lactobacillus* as the predominant species (Ravel et al., 2011, 2013; Gajer et al., 2012). Currently, more than ten CSTs are recognized depending on the experimental protocol; however, in general, six major CSTs derived from prevailing *Lactobacillus* species or anaerobic bacteria are widely accepted as defined by Ravel and collaborates (Ravel et al., 2011; van de Wijgert et al., 2014). Researchers have also described a diverse bacterial community (CST4) with a low proportion of lactobacilli and a higher vaginal pH in the healthy vagina largely in African American and Hispanic populations; this contrasted with Caucasian women, in which these anaerobic bacteria are usually associated with bacterial vaginosis (BV) (Ravel et al., 2011). A healthy vagina is dependent on estrogen production and the balance between highly adapted lactobacilli including resilience to disturbing conditions and local immunity (Farage et al., 2010; Gajer et al., 2012; Witkin et al., 2013). Internal (menstrual cycle) and external (antibiotics, intercourse, and intimate hygiene) factors can disrupt this balance and result in dysbiosis. When a dysbiotic state is established, the vagina becomes more vulnerable and prone to CST changes and infections caused by bacteria and yeasts. The highest transition potential is associated with the communities with the predominance of anaerobic bacteria and *L. iners* (Santiago et al., 2012; Lambert et al., 2013; Ravel et al., 2013; Brooks et al., 2017a). These changes take on importance when we consider that there is strong evidence that dysbiotic vaginal microbiota is connected with an increased risk of acquiring sexually transmitted agents (e.g. HIV, herpes simplex virus 2, chlamydia, gonococci or *Trichomonas*), more complications in reproduction health (infertility, preterm birth or miscarriage), and gynecological cancers (van de Wijgert and Jespers, 2017; Ventolini et al., 2022).

We hypothesized that CVD patients exhibit distinct bacterial profiles and CST dynamics depending on main microbial etiology compared to healthy controls and focused our research on the relationships between the composition of vaginal microbiome in CVD patients and community stability with respect to clinical status of a CST, i.e. affiliation to CVD or health conditions. The aim was to obtain information that would shed more light on the issue of dynamic change and function of the vaginal microbiota reflecting dysbiosis-driven pathology.

## 2 Materials and methods

### 2.1 Patients

This prospective cohort study was conducted between September 2014 and October 2018 in patients with CVD admitted to the Department of Obstetrics and Gynecology, University Hospital Hradec Kralove, Czech Republic. Only women aged 18–50 years were eligible for inclusion. Patients were excluded if they had any serious medical conditions such as pregnancy, cancer, diabetes mellitus, were taking antibiotic therapy or were currently undergoing immunosuppressive therapy to minimize confounding effects on microbiota and immunity.

CVD was defined as any vulvovaginal complaint based on a physical examination and personal history. As this broad definition of CVD is based on a few non-specific clinical criteria, CVD patients were divided into four main etiology groups at the time of the first (entry) visit based on microbiological findings and personal history: 1) women with unspecified discomfort usually corresponded with dysbiosis of vaginal microbiota characterized by poor clinical signs often not corresponding to a noticeable perception of subjective complaints. Other groups were CVD patients with 2) a yeast (vulvovaginal candidiasis, VVC) or 3) a bacterial etiology (BV or aerobic vaginitis); and 4) women with a mixed etiology of yeast and anaerobic bacteria, as it was difficult to decide which of these two etiologies was prevalent in the past or if both infectious agents participated in morbidity.

General medical history was collected from the patients enrolled in the study. Each participant was examined by a gynecologist and patients completed a detailed questionnaire. The initial gynecological examination included measurement of vaginal pH (pH strips, Merck) and microbiological investigation (NGS, microscopy, and culture) of vaginal fluid samples collected prior to the use of any antibiotics or local therapy. The patients were examined at a second (control) visit 4–8 weeks later when symptoms had resolved. The control group of healthy women underwent the same diagnostic procedures and specimen collection at both visits but without therapy during the follow-up period.

The study design was approved by the Ethics Committee of the University Hospital of Hradec Kralove (OHRP No. IORG0008813, approval No. 201408 S35). All women provided written informed consent.

### 2.2 Vaginal fluid analyses

Vaginal fluid samples were obtained using two Dacron polyester swabs, which were placed into the posterior fornix of the vagina for 20 s to achieve sufficient saturation. One sample was inserted into a polypropylene tube containing 1.5mL of phosphate-buffered saline, and the second was used for microbiological cultivation. The first aliquoted sample was shaken for 20 min and centrifuged at 300× g for 15 min at room temperature. The supernatant and pellets were isolated and stored in aliquots at -80°C until further analyses. The supernatant was used for additional microbiological cultivation, and the pellets were used for the extraction of bacterial DNA, real-time PCR, and NGS.

#### 2.2.1 Microbiology

Gram-stained vaginal smears from the posterior fornix of the vagina were examined under a microscope. The Nugent score was calculated according to the proportion of the main bacterial morphotypes (Nugent et al., 1991). Bacterial cultures were performed using blood agar, sheep blood agar, chocolate agar, Neisseria-selective agar, and MacConkey agar (BioMérieux CZ Ltd.) at 35–37°C under aerobic or 5% CO₂-enriched conditions. Anaerobes were cultivated using an anaerobic incubator.

Individual microbial isolates were identified by MALDI TOF mass spectrometry (Bruker Daltonics).

#### 2.2.2 Next generation sequencing

Microbiome analysis was based on NGS of bacterial 16S rDNA V4/V5 and fungal 18S rDNA internal transcribed spacer-1 (ITS1) variable regions. DNA was isolated from supernatant of vaginal tissue samples using QIAamp DNA Mini Kit (QIAGEN, Netherlands) according to the manufacturer’s instructions, with an elution volume of 200 µl. PCR amplification of the target regions was performed using the Q5 High-Fidelity polymerase (New England BioLabs, UK) according to the manufacturer´s protocol. In the first step, specific primers were employed; F519/R926 for V4/V5 16s rDNA [thermal profile 98°C for 30 s, 20x (98°C for 10 s; 70°C for 30 s; 72°C for 30 s), 72°C for 2 min] and 8F/ITS2 357R/ITS1F in the 18S-ITS1. PCR products were checked by agarose electrophoresis and purified by NucleoSpin^®^ Gel and PCR Clean-up (Macherey-Nagel, Germany) for NGS library preparation. The subsequent PCR reactions introduced sample-specific („barcode”) sequences, as well as Illumina P5 and P7 adapters required for the sequencing process. The Q5 High-Fidelity polymerase was employed with thermal cycling settings at: 98°C for 30 s, 15x (98°C for 10 s; 60°C for 30 s; 72°C for 30 s) and 72°C for 2 min. For ITS-1, the samples were indexed using a Nextera^®^ XT Index Kit (Illumina), according to the manufacturer’s protocol. Extended PCR products were purified from the agarose gel and quantified using Qubit and Qubit dsDNA HS Assay Kits (Thermo Fisher Scientific, USA). Equal amounts of indexed PCR products were pooled. The genomic library quality was assessed on an Agilent 2100 Bioanalyzer using an Agilent High Sensitivity DNA Kit (both from Agilent Technologies, USA), and quantity was assessed using a qPCR KAPA Library Quantification Kit (Kapa Biosystems, MA, USA). Library samples were mixed with the PhiX sequencing control (10-20%), denatured, and diluted to 8.5 pM. Paired-end sequencing was performed on an Illumina MiSeq sequencer using the MiSeq Reagent Kit v2–500 cycles (Illumina) following the manufacturer’s protocol. Microbiota analysis was performed on de-multiplexed and quality-filtered FASTQ data using the DADA2 pipeline (Callahan et al., 2016) and taxonomic categories were assigned using the RDP (trainset 16/release 11.5) and Silva (release 132) databases as references (Cole et al., 2014; Quast et al., 2013). Trimming parameters were set according to FIGARO application (Weinstein et al., 2019). Other parameters were left default as recommended by developers of DADA2. Data filtering, Alpha and Beta diversity, and the abundance of taxonomic categories were analyzed with R software 3.6, using PhyloSeq (McMurdie and Holmes, 2013).

CST classification was based on hierarchical clustering using the Jensen–Shannon divergence metric and Ward’s method, minimizing the total within-cluster variation for linkage analysis. Number of clusters was chosen according to CST classification previously published by Ravel et al. (2011).

As unstable CSTs, all labile and newly established CSTs were designated. Labile CSTs represented communities that subsequently changed during the follow-up period and were associated with CVD symptoms, while new CSTs were those resulting from labile CSTs at control visit.

#### 2.2.3 Quantification of *Lactobacillus crispatus* by real-time PCR

Nucleic acid was isolated from the pellets using tissue protocol of QIAamp DNA Mini Kit (Qiagen, Hilden, Germany). Detection of *L. crispatus* was performed by in-house real-time PCR. Primers and hydrolysis probe for *L. crispatus* were designed from 16S rRNA region to amplify a 150 bp amplicon (forward primer LC F35 GCG AGC GGA ACT AAC AGA TT, reverse primer LC R184 TGA TCA TGC GAT CTG CTT TC and probe labelled FAM-BHQ1 CTG CCC CAT AGT CTG GGA TA). The real-time PCR amplifications were performed on a Rotor-Gene Q instrument with the 25-μl reactions containing universal 2× gb IPC PCR Master Mix (Generi Biotech, Czechia) with internal positive control, primers both at a concentration 400 nM and dual labelled hydrolysis probes (FAM-BHQ1) at a concentration 200 nM. Primers and probes were synthesized from Generi Biotech (Czechia). Amplification parameters were as follows: 95°C for 5 min followed by 45 cycles, each of which comprised 95°C 15 s and 60°C for 30 s. PCR detection was performed as absolute quantification with standard curve generated with serial 10-fold dilution of linearized and normalized plasmid containing cloned target sequences for *L. crispatus* with concentration 10^7^ copies/µl (Generi Biotech, Czechia).

### 2.3 Statistical analysis

Statistical analysis was performed using a free software environment for statistical computing and graphics R1 version 4.4.2. The association between quantitative traits was evaluated by a two-tailed Mann-Whitney U test or *t*-test according to data distribution (normal or non-normal). The Shapiro-Wilk statistic was used to test for normality. Quantitative attributes were described as the mean and standard deviation of the mean, and asymmetric distributions were described as the median. Ordinal and nominal attributes were described as percentages with standard errors. Differences in the quantitative demographic characteristics between the CVD and healthy control groups were compared using *t*-test or Welch’s test (when normality was not rejected). If the normality of quantitative attributes was rejected, the Mann-Whitney U test was used to compare two independent samples. Comparisons of proportions were performed either with contingency table analysis (chi-square test with p-value either asymptotic or exact – Fisher or its variant Fisher-Freeman-Halton), based on frequencies.

Data on the abundance of individual taxa or study (sub)groups were presented as a proportion (%), determined by the number reads of a given taxon or taxon for a (sub)group divided by the total number of reads in the respective sample. A similar procedure was followed when paired tests were used (in-out analysis of CVD etiology groups). In the case of comparison of unpaired samples, abundance was calculated as the average of the averages of the number of reads for a given taxon or group.

Spearman’s rank correlation coefficient was used to analyze correlations between the variables investigated. The linear discriminant analysis (LDA) effect size (LEfSe) method was used to identify discriminative taxonomic markers using log LDA score cut-off of 2.0, followed by the Kruskal–Wallis test with a Wilcoxon test cut-off of p < 0.05. LEfSe analysis was conducted using bioconda package lefse (Segata et al., 2011).

In all analyses, p < 0.05 was considered statistically significant except for correlation analysis, in which more stringent criterion, p <0.01, was used in **Table 1** and **Supplementary Table S3** due to the small number of variables in some items.

**Table 1:** Baseline characteristics of patients with CVD and control group.

	Controls		CVD patients		P-value
	n (35)	%	n (91)	%	
Age (mean ± SD)	29.8 ± 7.19		31.2 ± 7.80		0.362*
Delivery	16	45.7	38	41.8	0.841§
Abortion	6	17.1	9	9.9	0.356§
HC	12	34.3	29	31.9	0.834§
IUD	5	14.3	11	12.1	0.769§
Allergy	8	22.9	29	32.2	0.385§
Smoking	4	11.4	7	7.8	0.500§
*Chlamydia* in anamnesis	1	2.9	11	12.1	0.177§
Frequency of episodes (per year)					< 0.0001#
0	35	100	0	0	
1 to 2	0	0	0	0	
3 to 4	0	0	3	3.3	
5 to 9	0	0	19	20.9	
10 to12	0	0	69	75.8	
Objective signs on entry visit					
overall	6	17.1	87	95.6	
discharge	20	57.1	82	90.1	< 0.0005§
erythema	0	0	4	4.4	0.575§
odor	1	2.9	19	20.9	0.076§
condyloma	0	0	3	3.3	0.373§
trauma	0	0	5	5.5	0.190§
other	0	0	13	14.3	0.011§
Number (per person)					< 0.001#
0	15	42.9	4	4.4	
1	19	54.3	62	68.1	
2	1	2.9	19	20.9	
≥ 3	0	0	24	26.4	
Subjective symptoms on entry visit					
overall	1	2.9	91	100	< 0.001§
discharge	1	2.9	79	86.8	< 0.001§
burning	0	0	20	22	0.001§
itching	0	0	68	74.7	< 0.001§
odor	0	0	12	13.2	0.016§
dyspareunia	0	0	10	11	0.033§
vulvodynia	0	0	5	5.5	0.190§
number (per person)					< 0.001#
0	34	97.1	0	0	
1	1	2.9	15	16.5	
2	0	0	52	57.1	
≥ 3	0	0	24	26.4	

HC, hormonal contraception; IUD, intrauterine device; § Fisher test, # Fisher-Freeman-Halton test, *Kolmogorov-Smirnov test.

## 3 Results

### 3.1 Study population groups

In total, 281 vaginal samples were obtained from 155 women. Of this initial sample size, 29 were further excluded because of unmatched criteria or the absence of a second sample during the control visit. For data evaluation and statistical analysis, 91 patients with CVD and 35 healthy controls were enrolled, corresponding to 182 and 70 samples, respectively. The age in both study groups was comparable with an average of 31.2 ± 7.80 years and 29.8 ± 7.11 years for CVD patients and control patients, respectively. All women were Caucasian.

The patients were distributed in CVD etiology groups and controls as follows: non-specific CVD group (n=24), yeast group (n=25), bacterial group (n=32), mixed group (n=10), and healthy controls (n=35).

Basic demographic data (**Table 1**) showed that most patients with CVD (83.5%) reported two or more complaints, but their increased perception of vulvovaginal discomfort did not correspond to the clinical picture; only 27.5% had two or more signs. Demographic features were not statistically significant between both groups except for majority of clinical signs and symptoms. Although not significant, compared to controls, patients with CVD tended to have more frequent allergy symptoms, a history of *Chlamydia* infection, and fewer abortions.

### 3.2 Vaginal pH and Nugent score

Compared to healthy controls, CVD patients had a higher Nugent score (mean ± SD 3.7 ± 1.78 vs. 3.4 ± 1.69, p=0.1269, t-test) and significantly higher vaginal pH (4.7 ± 0.61 vs. 4.5 ± 0.44; p=0.0120). When compared pH, it was significantly higher in CVD patients than controls only at entry visit (4.7 ± 0.56 vs. 4.5 ± 0.42, p=0.0286) not at control examination. Both the Nugent score and, to a lesser extent, vaginal pH were more affected by CST than by CVD etiology. The pH and Nugent score varied little between two visits within the CVD groups as well as CSTs; except for Nugent score in CSTs (median range 2.0 to 6.0).

With regard to CVD etiology, the control group and yeast etiology shared the lowest pH (median 4.4) and Nugent score (median 3.0), whereas the highest values were associated with the bacterial group (median pH 4.8, Nugent score 4.0). Within CSTs, CST1 (*L. crispatus* dominated) had the lowest pH and Nugent scores (median 4.4 and 2.0, respectively), whereas CST4 (diverse anaerobic bacteria) had the highest (median 5.0 and 6.0, respectively). The effect of sampling time on pH and Nugent score was negligible for all CSTs except for Nugent score in CST3 (*L. iners* dominated) the median of which significantly increased from 3.0 to 4.0 (p=0.0072).

### 3.3 Vaginal microbiota analysis

#### 3.3.1 Distribution of CSTs in healthy controls and CVD patients

The representation of CSTs in CVD patients and controls was as follows: CST1 (n=26), CST2 (n=15), CST3 (n=58), CST4 (n=23), and CST5 (n=4). Distribution of the CST categories was similar for both groups, but healthy controls lacked CST5 (*L. jensenii* dominated) and showed a higher proportion of CST1 than CVD patients (30.0% vs. 17.0%, p=0.0227, χ2 test) (**Supplementary Figure S1**). Furthermore, the proportion of CSTs was dependent on the clinical status with the opposite trend observed during follow-up period. While the proportion of CST3 decreased from 45.7% to 37.1% in healthy controls, that of CST4 increased from 17.1% to 22.9%. And vice versa in CVD patients, CST3 increased from 41.8% to 53.8% and CST4 decreased from 22.0% to 13.2%.

**Figure 1:**
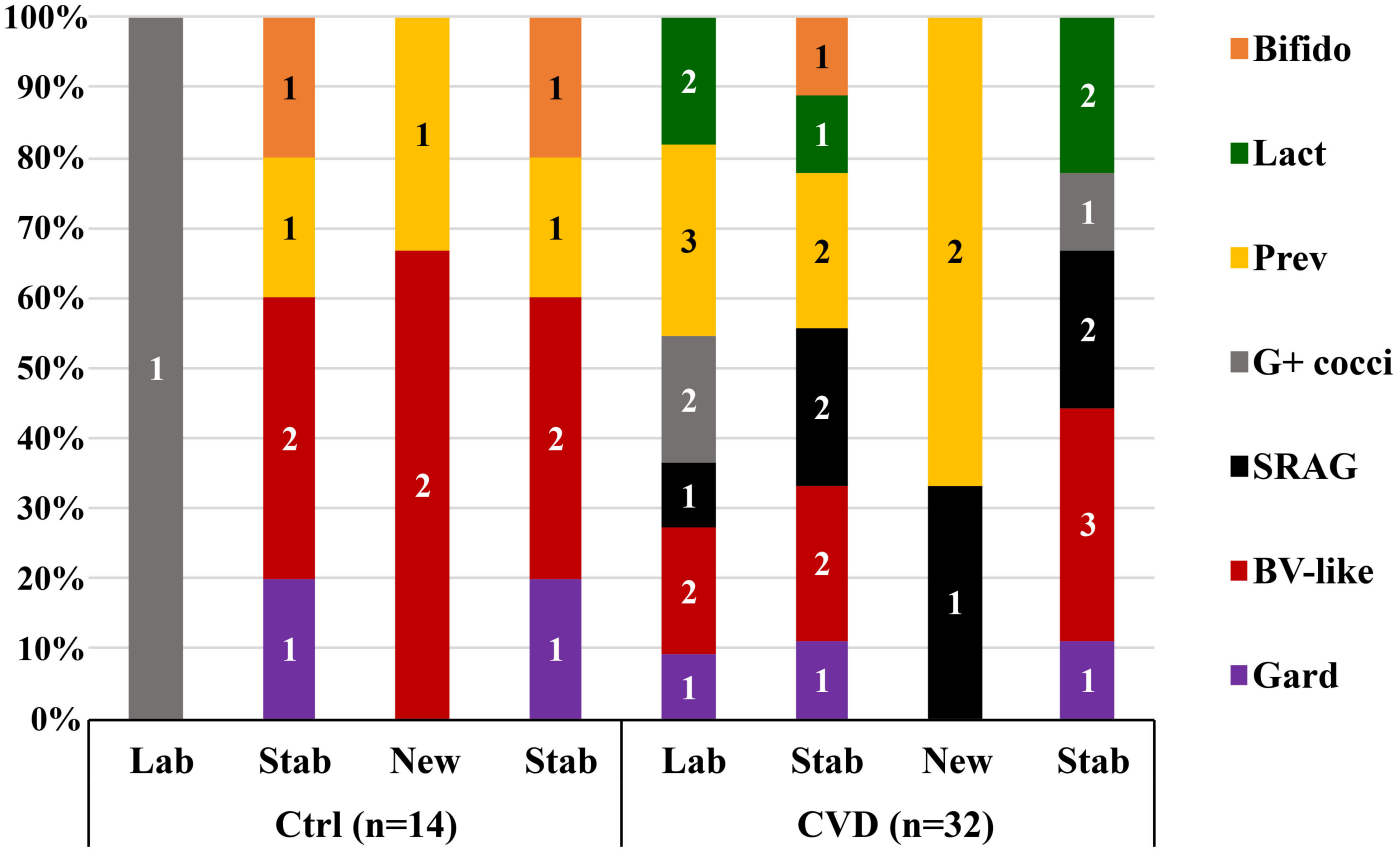
Distribution of unstable CST4 vaginotypes according to the predominant bacterial genus/species. Gard, *Gardnerella*; BV-like, *Atopobium*, *Sneathia*, *Megasphaera*; SRAG, *Streptococcus agalactiae*; G+ cocci, G-positive cocci (*Streptococcus* others 3×, *Enterococcus* 1×); Prev, *Prevotella*; Lact, *Lactobacillus*; Bifido, *Bifidobacterium*; Ctrl, control group; CVD, patients with CVD; Figures in columns represent a number of CSTs.

CST3 represented the most frequent vaginal community in all CVD groups, ranging from 37.5% (non-specific group) to 58.0% (yeast group) (**Supplementary Figure S1**). The proportion of CST1 was the largest in the control group (30.0%), the smallest in bacterial group (10.9%). CST2 (*L. gasseri* dominated) proportion was significantly higher in the non-specific group (29.2%) compared to other etiologies, including controls (p<0.036, χ2 test). The effect of sampling time was minimal; there was only a decrease in CST4 in the bacterial (from 34.4% to 21.9%) and non-specific groups (from 20.8% to 12.5%). The distribution of CST4 was similar in CVD and control group (17.6% vs. 20.0%), minimal in the yeast etiology (4.0%), and maximal in the bacterial group (28.1%). Seven subtypes of CST4 were described according to the relative abundance of a predominant species – five in controls and seven in CVD patients: 1) *Gardnerella*; 2) BV-like bacteria (*Atopobium*, *Megasphaera*, *Sneathia*); 3) *Streptococcus agalactiae*; 4) other G-positive cocci (3× non-*agalactiae* streptococci, 1× enterococci); 5) *Prevotella* species with dominance of *P. bivia*; 6) *Lactobacillus* spp. (3× *L. rhamnosus*, 2× *L. iners*), and 7) *Bifidobacterium* with dominance of *B. breve* (**Figure 1**; **Supplementary Figure S2**).

#### 3.3.2 Relative abundance of bacteria by a CST and CVD etiology

The composition of the vaginal microbiota of CVD patients and healthy controls was similar for majority of bacteria (**Supplementary Table S1**). More pronounced differences were evident at lower taxonomic levels especially in Actinobacteria - *G. vaginalis*, *A. vaginae* (today *Fannyhessea vaginae*), and bifidobacteria had significantly higher proportion in the control group than CVD patients [p=0.0003, p=0.0171, and p=0.0074, respectively, Mann-Whitney (MW) test]. The proportion of G-positive cocci in controls also exceeded the abundance in CVD patients, significantly only in the yeast group (p<0.0001), especially due to staphylococci. When compared lactobacilli expressed by more than 90% abundance of all reads per sample, they represented more than 50% of all samples in all CVD groups and healthy controls with maximum in yeast etiology (82.0% of samples). It corresponded with the maximum abundance of lactobacilli in the yeast etiology group (median 98.8%), which was significantly higher than that in the bacterial group (p<0.0042) and, on the contrary, was lower in BV-like bacteria, G-positive cocci, and *S. agalactiae* compared to both bacterial etiologies (p<0.0009, p<0.0007, and p<0.013, respectively) (**Supplementary Table S1**). The non-specific group displayed significantly higher proportion of *L. gasseri* than the other CVD etiologies and controls (median 0.099% vs. <0.034%, p<0.007). Other than Big Four lactobacilli were insignificantly more abundant in the microbiota of CST2 and partly CST4 (data not shown). As expected, CST4 bacteria significantly differed from the other CSTs (especially CST1 and CST3) due to the lower abundance of lactobacilli (median 4.66% vs. >95.7%, p<0.0001) and the higher of BV-like bacteria (p<0.0001), G-positive cocci (p<0.0023), bifidobacteria (p<0.0025), non-*bivia Prevotella* (p<0.001), and *Sneathia* (p<0.0009).

**Figure 2:**
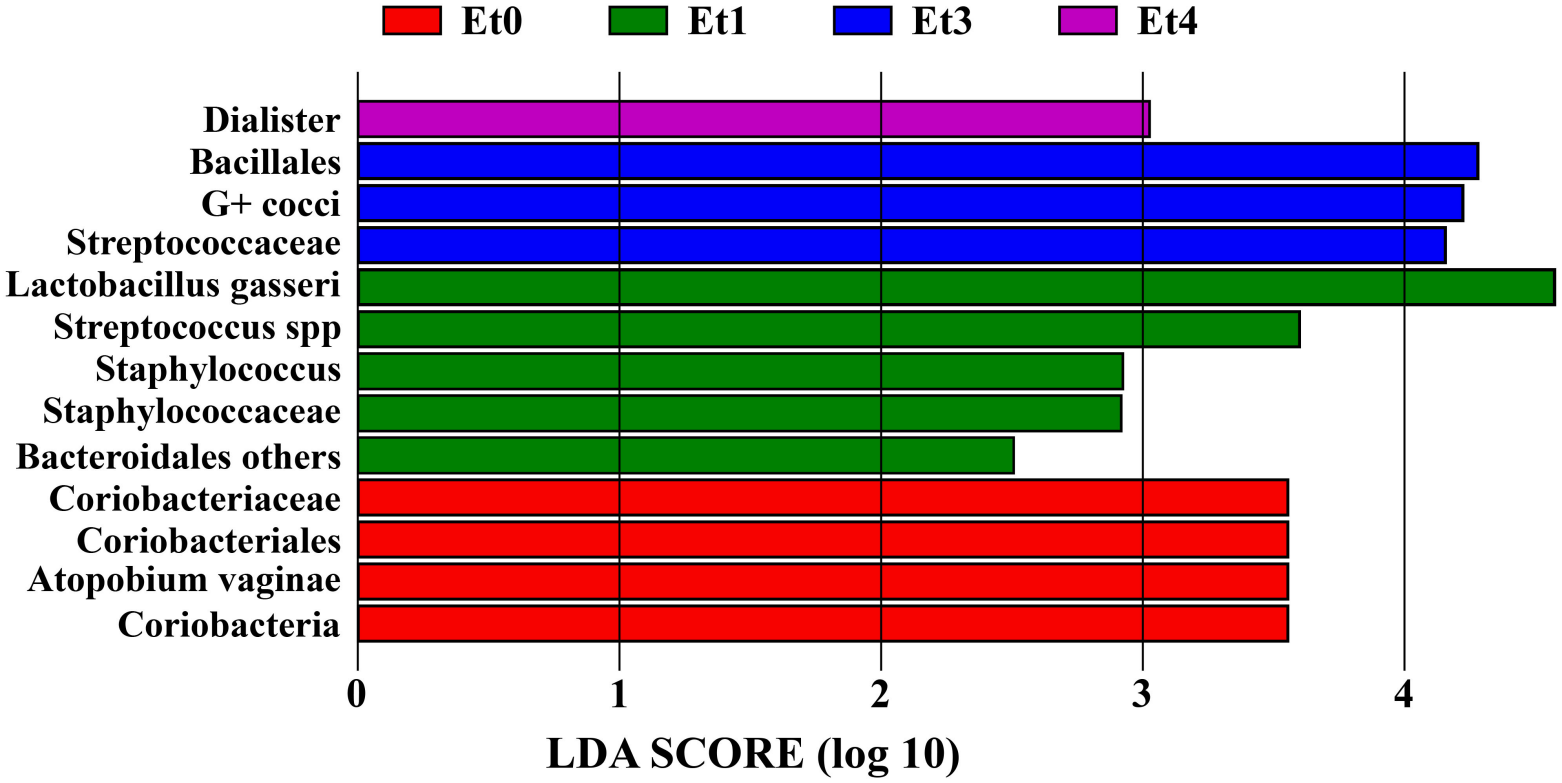
Linear discriminant analysis (LDA) score of CVD etiology groups and healthy controls. Et0 - healthy controls, Et1/Et3/Et4 - non-specific/bacterial group/mixed group of CVD patients, G+ cocci - G-positive cocci, LDA score - the higher value the stronger the associations between taxa and the respective group/taxon.

These results were in accordance with LDA (linear discriminant analysis) score that suggested non-specific group as the most distinct in CVD group due to a higher abundance of *L. gasseri*, staphylococci, streptococci, and some non-*Prevotella* Bacteroidetes; the same was true for G-positive cocci (mainly streptococci) in bacterial group and *A. vaginae* in healthy controls (**Figure 2**).

**Figure 3:**
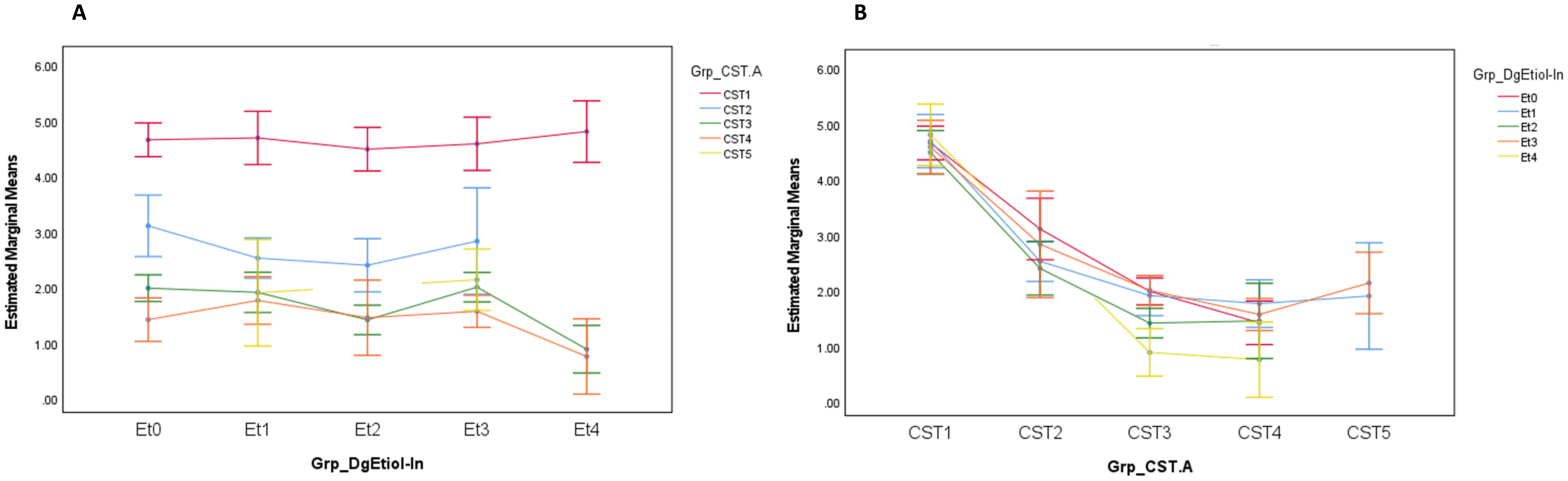
*Lactobacillus crispatus* load (DNA copies/mL) distribution by CVD etiology **(A)** and CST **(B)** at entry visit. Grp_DgEtiol - CVD group, Grp_CST - CST category, Et0/Et1/Et2/Et3/Et4 - healthy controls/non-specific/yeast/bacterial/mixed group of CVD patients.

The higher abundance of lactobacilli in CST1 and the yeast etiology group corresponded with the highest load of *L. crispatus* (1.1×10^9^ ± 1.87×10^9^ and 0.6 ± 1.72×10^9^ DNA copies/mL, respectively) among studied groups. At the same time, DNA load of *L. crispatus* was primarily dependent on CST and not on CVD etiology (**Figure 3**).

#### 3.3.3 Effect of clinical status

Microbiota composition differed by clinical status (health vs. CVD) in all CSTs. CST1 in the control group was associated with a higher proportion of *G. vaginalis* (p=0.0182, MW) and *A. vaginae* (p=0.0319) and a lower proportion of *S. agalactiae* (p=0.0417) compared to CVD cohort. In CST2, healthy controls showed a higher proportion of G-positive cocci (median 3.99% vs. 0.036%, p=0.0380), non-*agalactiae Streptococcus* (median 1.82% vs. 0.016%, p=0.0224), Clostridiales (p=0.0065), *Dialister* (p=0.0072), and a lower proportion of *A. vaginae* (p=0.0474) and *L. iners* (median 0.134% vs. 0.539%, p=0.0011). CST3 was comprised of significantly higher abundance of Firmicutes (p=0.0044), lactobacilli (p=0.0101), staphylococci (p<0.0001), and *G. vaginalis* (p=0.0382) in the controls and a higher abundance of Bacteroidetes (p=0.0225), especially non-*bivia Prevotella*, in patients with CVD (p=0.0055). In CST4, CVD patients showed a higher abundance of Firmicutes (median 40.9% vs. 26.5%, p=0.0734), specifically *L. gasseri* (median 0.037% vs. 0.006%, p=0.0287), and G-positive cocci (median 10.4% vs. 1.21%, p=0.2144), while healthy controls had a higher proportion of Actinobacteria (median 1.35% vs. 25.1%, p=0.0026), mainly bifidobacteria (median 0.002% vs. 4.35%, p=0.0048).

### 3.4 Stability of vaginal microbiota

#### 3.4.1 Distribution of unstable CSTs during the follow-up period

A total of 54 unstable CSTs (21.4%) were found in both cohorts, comprised of 27 labile CSTs at the initial visit and 27 new CSTs at the control visit, without a significant difference between CVD patients and healthy controls (23.1% and 17.1%, respectively, p=0.3038, χ2 test) (**Supplementary Figure S3**). The proportion of unstable (labile and new) CSTs fluctuated more by a CST (from 13.8% to 39.1%) than by a CVD etiology (from 16.0% to 20.0%) except for non-specific group (37%), which significantly differed from other CVD etiologies (p<0.027, χ2 test).

**Figure 4:**
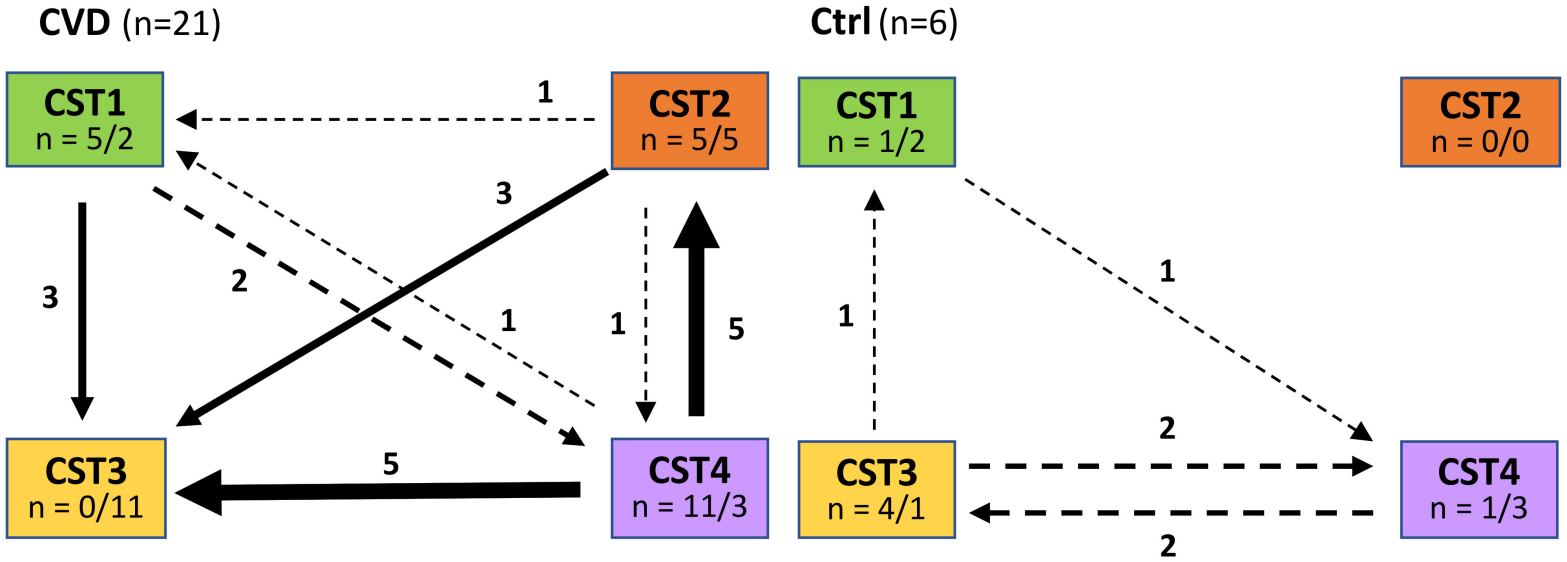
Comparison of CST transition between entry and control visit in patients with CVD and healthy controls. Ctrl, control group; CVD, CVD patients; n = x/y, x = number of labile CSTs, y = number of CSTs to which labile CSTs were converted.

Overall, CST2 and CST4 were associated with the greatest instability in CVD (**Figure 4**). The proportion of labile CST3 was significantly lower (p<0.001, χ2 test) from other CSTs (CST5 not involved) in CVD groups and CST3 represented the most stable CST. CST3 was the community to which labile CSTs converted most often, while in the control group, CST3 was most labile CST with a preferential shift to CST4. The opposite trend was observed for CST2 with unstable CSTs only in CVD patients, but this may have been influenced by the low number of women with CST2 (n=3) in the controls. In addition, CST2 showed no difference in the proportion of labile and new CSTs in contrast to other CSTs.

#### 3.4.2 Vaginal microbiota of stable and unstable CSTs

##### 3.4.2.1 CST stability affected microbiota composition more in CVD patients than control group

In general, the stability of CST affected the bacterial abundance more than sampling time (**Supplementary Table S2**). The effect of sampling time was observable only in CVD with significantly more BV-like bacteria in labile CSTs than new CSTs (p=0.0261, Wilcoxon paired sample test), and vice versa for *L. iners* (p=0.0065).

**Figure 5:**
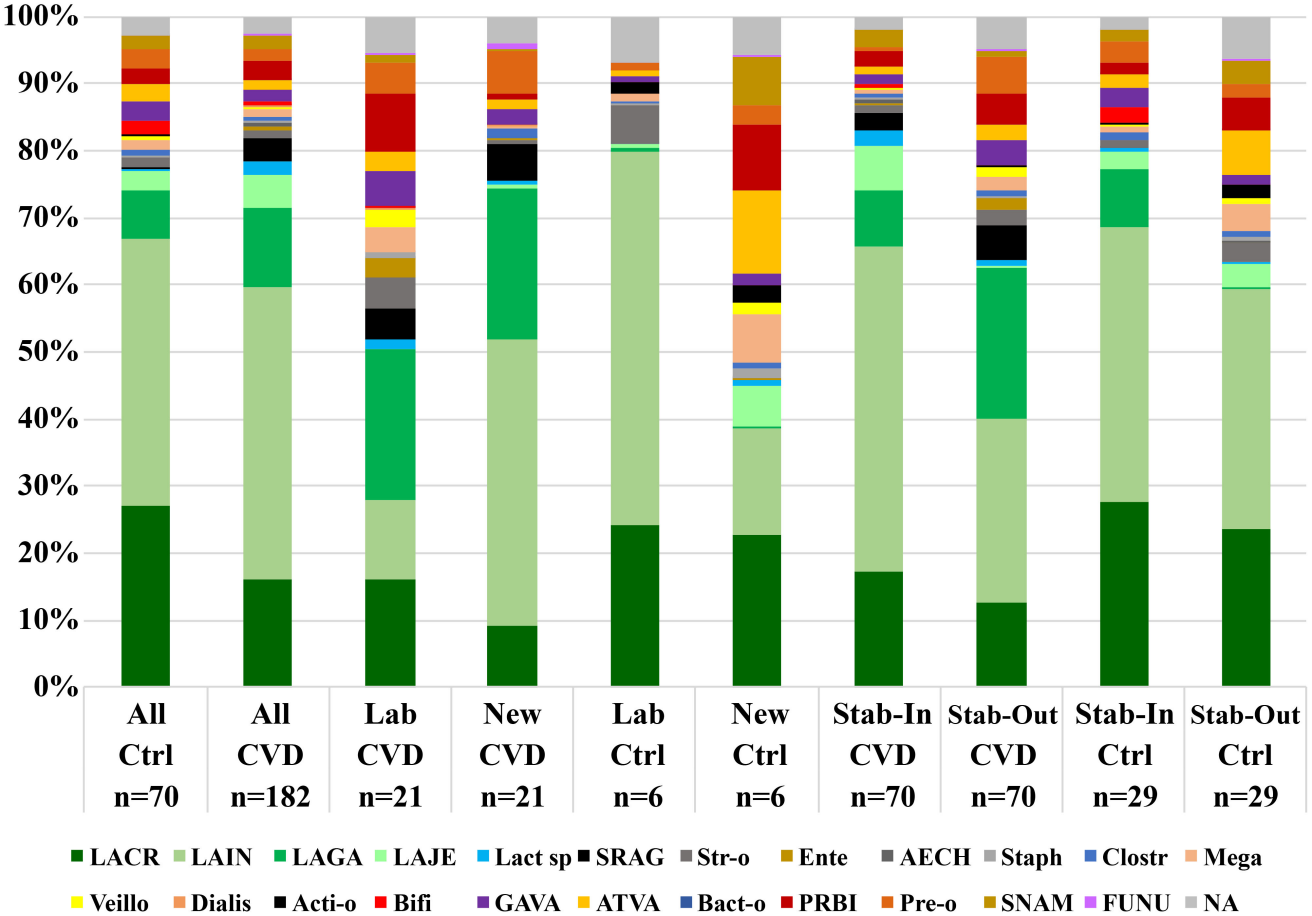
Comparisons of the vaginal microbiota composition of unstable and stable CSTs in and between CVD patients and healthy controls. Ctrl, control group; CVD, CVD patients; Lab, labile CSTs; New, new CSTs; Stab-In/Out, stable CSTs at entry/control visit; LACR, *Lactobacillus crispatus*; LAIN, *L. iners*; LAGA, *L. gasseri*; LAJE, *L. jensenii*; Lact sp, *Lactobacillus* spp. (except for LACR, LAIN, LAGA, and LAJE); SRAG, *Streptococcus agalactiae*; Str-o, streptococci other than SRAG; Ente, *Enterococcus* spp.; AECH, *Aerococcus christensenii*; Staph, *Staphylococcus* spp.; Clostr, Clostridiales; Mega, *Megasphaera* spp.; Veillo, *Veillonella* spp.; Dialis, *Dialister* spp.; Acti-o, other Actinomycetales; Bifi, Bifidobacteriales; GAVA, *Gardnerella vaginalis*; ATVA, *Atopobium vaginae*; Bact-o, other Bacteroidales; PRBI, *Prevotella bivia*; Pre-o, *Prevotella* spp. other than *bivia*; SNAM, *Sneathia amnii*; FUNU, *Fusobacterium nucleatum*; NA, other bacteria (not identified).

When labile or new CSTs were compared with stable CSTs, more significant differences were noted in CVD patients than controls (**Supplementary Table S2**). Labile CSTs of CVD group had significantly higher abundance of Actinobacteria, Bacteroidetes, Fusobacteria G-positive cocci (mainly non-*agalactiae* streptococci), BV-like bacteria (incl. *Prevotella*, *Veillonella*, and *Megasphaera*), *L. gasseri*, and bifidobacteria (for all p<0.010, MW), while stable CSTs of CVD patients showed a higher proportion Firmicutes, lactobacilli, and *L. iners* than labile CSTs (p<0.025). Similar results were obtained by comparing new and stable CSTs in the CVD group. In healthy controls, the difference between unstable and stable CSTs was generally less frequent and significant only between new and stable CSTs. Actinobacteria, staphylococci, *Prevotella* (especially non-*bivia* species), and BV-like bacteria (especially *Veillonella*) showed a higher proportion in new CSTs (p<0.050), Firmicutes and lactobacilli a higher proportion in stable CSTs (p<0.033) (**Figure 5**; **Supplementary Table S2**).

##### 3.4.2.2 Clinical status affected microbiota composition and CST stability in CVD patients and control group

The comparison of stable CSTs between both cohorts revealed significantly lower abundance of G-positive cocci (p=0.0133), especially staphylococci (p=0.0003, MW), *G. vaginalis* (p=0.0017), *A. vaginae* (0.0253), and bifidobacteria (p=0.0044) in CVD than control group (**Supplementary Table S2**). As regards unstable CSTs, *L. gasseri* and *S. agalactiae* were a higher proportion in CVD than control group (p=0.0469 and p=0.0100, respectively), while *G. vaginalis* showed a higher abundance in healthy controls (p=0.0424).

Comparing unstable CSTs of both main groups showed a noticeable opposite trend depending on clinical affiliation – labile CSTs of CVD group were associated with a lower abundance of Firmicutes and lactobacilli (specifically *L. crispatus* and *L. iners*), and with a higher proportion of non-*bivia Prevotella*, BV-like bacteria, and *L. gasseri* compared to controls, and vice versa in new CSTs for Firmicutes, lactobacilli (and *L. iners*), BV-like bacteria, and non-*bivia Prevotella*. In addition, new CSTs of CVD patients showed Actinobacteria, staphylococci, and *P. bivia* a significantly lower abundance than control group (p=0.0227, p=0.0259, and p=0.0064, respectively) (**Supplementary Table S2**).

##### 3.4.2.3 Comparison of unstable and stable CSTs by a CST and CVD group

Intergroup comparison of stable and unstable CSTs revealed the difference between microbiota only in CST3 for several bacterial taxa and groups – unstable CSTs were characterized by higher proportion of G-positive cocci, Actinobacteria (bifidobacteria), *Prevotella*, and Fusobacteria (*S. amnii*), while lower abundances were connected Firmicutes, lactobacilli, especially *L. iners* (p=0.0255, MW). In contrast, *L. iners* abundance was significantly higher in unstable than stable CSTs of CST1 (p=0.0093) (data not shown).

In case of CVD etiologies, the differences between unstable and stable CSTs were noticeable in all groups, least in the control group, only for Actinobacteria, and most in non-specific group with a similar profile such as CST3, in addition, with higher proportion of *S. agalactiae* in unstable CSTs compared to stable ones (p=0.0265, MW) (data not shown).

### 3.5 Diversity of vaginal microbiota

In general, both healthy controls and CVD patients were characterized by significantly higher α-diversity of unstable than stable CSTs (**Supplementary Figure S4**). CST categories showed more variability of α-diversity than CVD groups (**Supplementary Figure S5**). The lowest α-diversity was associated with CST3 and CST2, the highest with CST4 and CST1. In CVD cohort, α-diversity was minimal in yeast and non-specific etiology, while in bacterial group it was maximal.

### 3.6 Correlation analysis

In general, Spearman´s analysis revealed that the correlation profile in healthy controls and CVD patients was similar expressed by cumulative correlation coefficient (ccc=48.06 vs. 44.77), but important differences were evident in the details (**Table 2**; **Supplementary Table S3**). In CVD group, ccc for the Big Four *Lactobacillus* species was negative (ccc=-0.335), in controls was positive (ccc=0.650) (**Supplementary Table S3**). On the other hand, both main study groups were characterized by a strong negative correlation between *L. crispatus* and *L. iners*, but *L. crispatus* was positively correlated with non-Big Four lactobacilli, while *L. iners* did negatively. In addition, the ccc for the Big Four lactobacilli with other bacteria was more negative in healthy controls than that in CVD group (ccc=-16.6 vs. -3.38), while ccc among non-*Lactobacillus* bacteria was more positive in controls than CVD patients (ccc=60.0 vs. 44.9). Furthermore, both cohorts shared a negative correlation between *L. iners* and G-positive cocci especially non-*agalactiae* streptococci and staphylococci.

**Table 2:** Spearman correlation coefficient between main bacterial taxa in patients with CVD and control group.

Ctrl	In										
Out	Firm	Acti	Bact	Fuso	Prot	Tene	NA	Lact	Cocci	Prev	BV-like
Firmicutes		**-0.643**	**-0.755**	*-0.394*	-0.244	*0.399*	**-0.861**	**0.988**	**-0.719**	**-0.758**	**-0.859**
Actinobacteria	**-0.834**		**0.470**	0.189	0.276	-0.107	0.333	**-0.692**	**0.480**	**0.482**	**0.723**
Bacteroidetes	**-0.871**	**0.604**		**0.527**	0.247	-0.212	**0.752**	**-0.744**	**0.722**	**0.993**	**0.794**
Fusobacteria	-0.309	0.219	**0.469**		0.250	-0.265	**0.496**	*-0.375*	0.305	**0.544**	*0.362*
Proteobacteria	*-0.425*	*0.423*	*0.351*	-0.120		-0.177	0.236	-0.271	*0.396*	0.246	0.278
Tenericutes	-0.190	0.247	0.075	0.156	0.269		*-0.409*	*0.388*	-0.256	-0.212	-0.246
NA	**-0.793**	**0.530**	**0.708**	0.248	0.153	0.103		**-0.826**	**0.689**	**0.747**	**0.673**
*Lactobacillus*	**0.971**	**-0.863**	**-0.843**	-0.295	**-0.461**	-0.175	**-0.722**		**-0.743**	**-0.748**	**-0.901**
G+ cocci	**-0.672**	**0.655**	**0.609**	0.168	**0.464**	0.065	**0.524**	**-0.756**		**0.735**	**0.777**
*Prevotella*	**-0.855**	**0.592**	**0.995**	**0.484**	*0.359*	0.082	**0.693**	**-0.831**	**0.621**		**0.804**
BV-like	**-0.887**	**0.844**	**0.829**	0.295	*0.422*	0.076	**0.566**	**-0.941**	**0.763**	**0.821**	
CVD	In										
Out	Firm	Acti	Bact	Fuso	Prot	Tene	NA	Lact	Cocci	Prev	BV-like
Firmicutes		**-0.613**	**-0.670**	**-0.328**	-0.028	0.101	**-0.881**	0.947	-0.546	-0.674	-0.794
Actinobacteria	**-0.590**		**0.376**	*0.254*	0.021	-0.130	**0.381**	-0.573	0.367	0.369	0.534
Bacteroidetes	**-0.622**	**0.391**		**0.518**	0.134	-0.006	**0.560**	-0.629	0.489	0.995	0.713
Fusobacteria	**-0.368**	*0.211*	**0.509**		0.113	*-0.247*	**0.309**	-0.355	0.466	0.496	0.451
Proteobacteria	*-0.257*	0.013	**0.292**	*0.245*		-0.033	0.020	-0.107	0.288	0.145	0.147
Tenericutes	0.025	-0.146	0.123	-0.169	0.013		-0.102	0.121	*-0.254*	0.002	-0.159
NA	**-0.843**	**0.311**	**0.513**	**0.341**	**0.296**	0.022		-0.869	0.598	0.571	0.687
*Lactobacillus*	**0.944**	*-0*.*547*	**-0.578**	**-0.332**	*-0.261*	0.057	**-0.828**		**-0.665**	**-0.633**	**-0.874**
G+ cocci	**-0.551**	**0.376**	**0.421**	**0.341**	0.154	-0.124	**0.596**	**-0.663**		**0.490**	**0.760**
*Prevotella*	**-0.627**	**0.383**	**0.997**	**0.512**	**0.292**	0.126	**0.521**	**-0.584**	**0.421**		**0.709**
BV-like	**-0.798**	**0.522**	**0.647**	**0.404**	*0.236*	0.021	**0.672**	**-0.887**	**0.729**	**0.644**	

Bolded = p<0.01, italic = p<0.05.

NA - other bacteria (not identified); In/Out - entry/control visit.

G+ cocci, G-positive cocci (strepto/staphylo/entero/aero-cocci), BV-like = G-positive cocci, Clostridiales, *Veillonella*, *Megasphaera*, *A. vaginae*, *G. vaginalis*, bifidobacteria, *Prevotella*, *Fusobacterium, Sneathia*.

Heatmap-like visualization - the color highlight represents the size of cc (the darker the green the lower the cc; the darker the orange the higher the cc).

Firmicutes in both main cohorts were negatively correlated with other bacterial taxa (most with BV-like bacteria), it contrasted with positive correlations among the main non-Firmicutes groups (**Table 2**). The exception was lactobacilli with a strong positive correlation (cc>0.940) with most contribution of *L. iners* (cc>0.360). These results corresponded with a positive correlation between vaginal pH and Nugent score for the control (cc>0.315) and CVD (cc>0.385) group, in addition, both markers negatively correlated with *Lactobacillus* abundance and positively with G-positive cocci, Actinobacteria, and Bacteroidetes. Detailed analysis revealed that *L. iners* was responsible for this negative correlation, on the contrary, *L. gasseri* positively correlated with G-positive cocci, more in CVD group than controls (data not shown).

The correlation (ccc) was also affected by CST stability depending on clinical status (**Supplementary Figure S6**). While the ccc for non-lactobacilli among themselves was strongly positive in both cohorts (more in controls) for both stable and unstable CST, the ccc for lactobacilli vs. non-lactobacilli was negative for the control group and slightly positive for CVD patients with small difference between stable and unstable CST in contrast to the ccc for lactobacilli, which was positive for unstable CST and very weakly negative for stable CST in both groups. Furthermore, evaluation of the proportion of individual lactobacilli showed that the Big Four lactobacilli increased the negative ccc between lactobacilli and non-lactobacilli, while the other (non-big Four) lactobacilli contributed to the positive ccc in lactobacilli of both main cohorts (data not shown).

In the CVD groups, ccc for non-lactobacilli among themselves was highly positive for unstable and stable CSTs in all etiologies (**Supplementary Figure S7**). When *Lactobacillus* species were evaluated, the ccc of unstable CSTs was positive in all CVD etiologies, and negative in stable CSTs, except for the bacterial group with positive ccc. A comparison of ccc for lactobacilli vs. non-lactobacilli showed largely negative values for both stable and unstable CSTs of majority groups except for non-specific etiology with positive ccc for unstable CSTs.

In CSTs, the ccc for other bacteria than lactobacilli was positive for stable and unstable CSTs in all CSTs (**Supplementary Figure S7**). The ccc for lactobacilli was weakly negative in the stable and unstable CSTs of CST1 and CST3, and positive for the unstable and stable CSTs of CST2 and CST4. When lactobacilli and non-lactobacilli were compared, CST2 and CST4 shared negative ccc for stable CSTs, while CST1, CST2, and CST3 displayed positive ccc for unstable CSTs. The ccc of unstable CSTs of CST4 was negative. Furthermore, CST2 was the only CST where *L. crispatus*, *L. iners*, and *L. gasseri* were negatively correlated with G-positive cocci, whereas in the other CSTs, G-positive cocci were negatively correlated with only one of the three lactobacilli. In addition, the correlation between *L. gasseri* and *L. crispatus* was positive in CST3 (cc>0.350) and CST4 (cc>0.490), but slightly negative (cc<-0.160) for CST1 and no correlation for CST2 (data not shown).

Spearman’s analysis also suggested differences in the correlation profiles of CST1 and CST3 compared to those of CST2 and CST4 (CST5 was omitted because it included only four patients). CST1 and CST3 shared negative ccc for lactobacilli in contrast to positive in CST2 and CST4. CST4 differed from other CSTs much lower total ccc (ccc=12.9 vs. ≥42.0). CST1 and CST3 displayed a more negative correlation between Firmicutes and G-positive cocci than CST2 (weakly negative), whereas in CST4, the correlation was positive (cc>0.480). This change corresponded with negative correlations between lactobacilli and G-positive cocci in all CSTs (cc<-0.345), except for CST4 (cc>-0.020). The correlation between G-positive cocci and *Prevotella* species was positive for CST1 and CST3 (cc>0.375), weakly negative for CST2 (cc<-0.035), and more negative for CST4 (cc<-390).

## 4 Discussion

The results of our study confirmed the pivotal role of lactobacilli in the vaginal environment and showed that some changes in the microbiota were closely related to the clinical status (healthy vs. CVD conditions) and stability of the CST (Ravel et al., 2011; France et al., 2022). As expected, the Nugent score and vaginal pH were higher in patients with CVD than in healthy controls, with the Nugent score being more dependent on the CST than on CVD etiology and sampling time. Both markers were positively correlated each other and showed a negative correlation with lactobacilli and a positive one with G-positive cocci and BV-like bacteria (mainly *Prevotella* spp.). Moreover, there was an opposite trend in the dynamics of both parameters in the microbiota during the follow-up period; while pH tended to improve (mainly in bacterial etiologies and CST2 and CST4) at the control visit, Nugent score tended to worsen (especially in the non-specific and yeast etiologies and in all CSTs except for CST1). This contradiction could be due to both therapeutic intervention at entry examination in CVD patients, the different proportions of bacteria associated with the construction of the Nugent score, and the factors responsible for acidic vaginal pH. By definition, *L. crispatus* and other lactobacilli participate in the calculation of the Nugent score together with bacteria, such as *Gardnerella, Bacteroides*, and curved G-negative rods (Nugent et al., 1991). In contrast, the vaginal pH can be influenced by the metabolic activity of a broad spectrum of bacteria, yeasts, and vaginal epithelial cells (Linhares et al., 2011). This suggests that pH is a more complex and that changes in the vaginal microbiota may not be always specifically associated with changes in pH, likely due to the contribution of redundant bacteria other than *Lactobacillus* species and epithelial cells to vaginal acidity.

In general, differences in the abundance of vaginal bacteria between CVD group and healthy controls were most evident in lactobacilli and BV-like bacteria. Patients with CVD tended to have a higher relative abundance of *L. iners* at the expense of *L. crispatus*, whereas the opposite trend was observed for controls, which corresponded to a negative correlation between *L. crispatus* and *L. iners* in both study groups. However, a closer look suggested that the abundance of some bacteria was associated with CST stability and showed an opposite trend during the microbiota transition in the CVD versus control group – that is, in CVD patients, the microbiota of unstable CSTs was disrupted at the entry visit (in labile CSTs) while in healthy controls at the control visit (in new CSTs). For example, the proportion of *L. iners* in the labile CSTs was significantly higher in the control group than in the CVD group (median 71.2% vs. 0.86%), the opposite was true for both new and stable CSTs. In contrast, *G. vaginalis* displayed a different pattern related only to clinical status, that is, its abundance was higher in healthy controls than in patients with CVD, regardless of stability status. As *G. vaginalis* is commonly involved in BV, this contradiction can reflect the existence of *G. vaginalis* strains with variable pathogenic adaptations and virulence factors (e.g., cohesivity to epithelia, biofilm, or toxin production), as postulated for different genomospecies in line with the concept of metagenomic subspecies (Machado and Cerca, 2015; Schellenberg et al., 2017; Ma et al., 2012; Holm et al., 2023). Such adaptation may determine whether a given strain will contribute to dysbiosis/BV or to the build-up of the vaginal milieu as an alternative lactate producer. The latter is suggested by the distribution of *G. vaginalis* in our CST4 between CVD and controls and even more so between stable and unstable CSTs (**Figure 1**; **Supplementary Figure S2**).

Intergroup evaluation of the CVD microbiota showed different dynamics of individual lactobacilli by CVD etiology. The *L. gasseri* abundance was significantly higher in non-specific group than that of the other etiologies. The proportion of *L. crispatus* and *L. jensenii* was similar across all CVD groups and this was also true for *L. iners* although this species tended to be more abundant in yeast etiology. Further, correlation analysis revealed a different nature of interrelationships among the Big Four lactobacilli depending on the clinical status and CST stability. *Lactobacillus iners* negatively correlated with the other Big Four species, most with *L. crispatus* (more in stable than unstable CSTs) in both study groups. In contrast, the correlations between *L. crispatus* and *L. gasseri* (or *L. jensenii*) were positive as well as between *L. gasseri* and *L. jensenii*. These results suggested a largely antagonistic relationship between *L. iners* and other formative lactobacilli in the vagina and/or a better adaptation potential of this species to disturbing conditions (Zheng et al., 2021). In general, *Lactobacillus* abundance fluctuated with an opposite trend in unstable CSTs, while in the CVD group it increased at control visit, in healthy controls it decreased (**Figure 5**). These changes in the microbiota were accompanied with an increase in the proportion of CST3 (*L. iners*) in CVD patients and a decrease in healthy controls during follow up and corresponded with manifestation of clinical symptoms of CVD at the entry examination. It suggests that reduced abundance of *L. iners* in CVD led to the disbalance of the vaginal microbiota and dysbiosis accompanied by CVD symptoms, whereas an increased proportion of *L. iners* (similar to stable CSTs) was associated with remission. It contrasted with healthy controls where the decreased proportion of *L. iners* was connected to control visit when most dysbiotic changes of microbiota occurred. This is in line with the current view of *L. iners* and its ambivalent role for vaginal health (Petrova et al., 2017). Unlike *L. iners* and CST3, *L. gasseri* and CST2 showed a different dynamic and dependency. CST2 was distinguished by significantly higher proportion in non-specific group than other CVD etiologies, and at the same time, non-specific group, together with CST4, shared the highest proportion of labile CSTs. In addition, all new CST2 came from labile CST4. Our results suggested that *L. gasseri* may indicate CST instability in CVD patients closely related to non-specific group as a result of adaptation to CVD conditions, which is in contrast to healthy women in whom CST2 rarely transited to another CST (Verstraelen et al., 2009; Gajer et al., 2012). Interestingly, there is a resemblance of CST2/CST4 microbiota in CVD to grade Ib microbiota with a predominance of *L. gasseri* and *L. iners* in pregnant women based on Gram staining (Verhelst et al., 2005; Verstraelen et al., 2009). In summary, it cannot be said unequivocally if *L. gasseri* or CST2 represent a specific signature of the vagina of CVD patients; therefore, more robust data are needed; however, our results suggest an association of *L. gasseri* with dysbiotic conditions and community instability at least for the subgroup of CVD patients with non-specific microbiota, which is consistent with some previous findings (Verstraelen et al., 2009; Witkin et al., 2013; Brooks et al., 2017a).

Another Big Four *Lactobacillus*, *L. jensenii*, tended to have a higher abundance, apart from CST5, in CST1 and CST3 than in CST2 and CST4. Like *L. crispatus*, *L. jensenii* is considered to be an indicator of vaginal health even when it is a weaker lactic acid producer compared to *L. crispatus* (Ravel et al., 2011). Therefore, CST5 is more likely to be expected in healthy controls than in CVD patients (Antonio et al., 1999; Martinez et al., 2008; Ravel et al., 2011; Witkin et al., 2013). Only four CST5 were observed in our patients with CVD and its absence in the controls was a bit surprising but it is consistent with the description of lower colonization strength of *L. jensenii* (Verstraelen et al., 2009). On the other hand, NGS analysis revealed a lower abundance of *L. jensenii* in controls than in CVD.

Other than the Big Four lactobacilli (mainly *L. rhamnosus*, *L. frumenti*, *L. delbrueckii*, and *L. vaginalis*) showed a higher prevalence in CST2 and CST4 and minimal differences among CVD groups except for *L. rhamnosus* with more abundance in bacterial etiology than others, which suggested a possible role of alternative *Lactobacillus*-derived microbiota in dysbiotic vagina or decreased susceptibility to antibiotics used for the treatment of BV.

In contrast to lactobacilli, G-positive cocci predominated in the CVD cohort over controls, in CST4 over the other CSTs, and in unstable over stable CSTs. Our results revealed seven subtypes of CST4 with approximately even distribution between CVD patients and controls (**Figures 1**, **S2**), which corresponded to more than 5% abundance of G-positive cocci per sample in 59.4% of patients with CVD versus 28.6% of the control group. Specifically, *S. agalactiae* predominated in CVD patients with CST4 (>32% abundance in 21.9% of CST4s) compared to healthy controls with CST4 (overall <0.03% abundance). The closer relationship between CST4 and *S. agalactiae* (and other G-positive cocci) was underscored by the fact that in non-CST4 groups there were only six women (two controls and four CVD patients) with *S. agalactiae* abundance >1% (four of the six women belonged to CST2). Further, there was a trend of higher proportion of *S. agalactiae* in labile CSTs in the CVD group compared to controls, whereas in controls, almost all cocci in labile CSTs belonged to non-*agalactiae* streptococci. It suggests that *S. agalactiae* in CVD patients contributes to instability of the CST and possibly to the promotion of CVD episodes, whereas in healthy controls, other streptococci may represent a compensatory mechanism for the loss of lactobacilli and lactate production. The increased abundance of *S. agalactiae*, enterococci and staphylococci suggest a closer relation of CST2 to aerobic vaginitis (AV), while in CST4, in addition to the increased proportion of the above-mentioned bacteria, a higher abundance of *G. vaginalis*, *A. vaginae* and *Megasphaera* spp. was evident, which is more indicative of a chronic form of BV (Kaambo et al., 2018). Regarding *S. agalactiae*, it is important to mention the described relationship of this coccus to invasive Group B *Streptococcus* infection in newborns and, together with enterococci, also as a risk factor for delivery complications (Kaambo et al., 2018). The minimal proportion of CST4 and *S. agalactiae* in the yeast group and healthy controls, together with the highest relative abundance and load of *L. crispatus* in CST1/CST3 may reflect more specific relationships between lactobacilli and yeasts in the vagina, which in patients with higher yeast burden more likely corresponds with recurrent vulvovaginal candidiasis (data not shown) (Rönnqvist et al., 2006; Brown et al., 2019; Novak et al., 2022; Sobel and Vempati, 2024; Song et al., 2024).

*Prevotella* species, especially non-*bivia Prevotella*, represent another group of bacteria closely related to CST4, with a generally recognized negative impact on vaginal health through metabolic products, which positively affect biofilm production or the growth of some BV-like bacteria (Aroutcheva et al., 2008; Ravel et al., 2011; Pybus and Onderdonk, 1997, 1998, Si et al., 2017; George et al., 2024; Sousa et al., 2024). In our study, *Prevotella* spp. shared largely positive correlations with other BV-like bacteria (G-positive cocci, *Gardnerella*, *Megasphaera*, *Atopobium*, *Sneathia*) in all CSTs, with the highest abundance in CST4 being significantly higher than that in other CSTs and, on the contrary, was negatively correlated with lactobacilli. This is in line with the co-occurrence or cooperation of *Prevotella* with *G. vaginalis* and other BV-like bacteria in the pathogenesis and recurrence of BV (Pybus and Onderdonk, 1997, 1998, Si et al., 2017; Plummer et al., 2023; Sousa et al., 2024). At the species level, the higher abundances of *P. amnii* and *P. timonensis* in CST4 differed the most from the other CSTs (data not shown). *Prevotella timonensis* is considered the main producer of sialidase that degrades the protective mucin layer in the vagina, which supports the generally accepted negative contribution of *Prevotella* to vaginal dysbiosis and pathology (Pelayo et al., 2024; George et al., 2024). On the other hand, our results showed the highest abundance of *P. timonensis* in the controls and the non-specific etiology. Furthermore, there was a generally higher proportion of *Prevotella* in the control group compared to the CVD group in all CSTs except for CST3, and *Prevotella* species positively correlated with BV-like bacteria in healthy controls, suggesting a more complex role of *Prevotella* in health versus disease in the vagina. This trend may depend on the given *Prevotella* species, the context of another microbiota, and vaginal physiology.

Although CST3 (*L. iners* dominated) was the most common CST in both healthy controls and CVD patients, there was a striking difference in CST stability between the two study groups. While CST3 was shown to be the most stable CST in the CVD groups, in healthy controls, it was the most labile, in line with the findings of previous studies (**Figure 4**) (Jakobsson and Forsum, 2007; Kalra et al., 2007; Macklaim et al., 2011; Petrova et al., 2015). This supports the view that *L. iners* has the ability to adapt to various unfavorable and transient conditions, while *L. crispatus* acts as a pro-health factor through the production of D-lactate, bacteriocins, and H_2_O_2_, which suppress potentially harmful anaerobic bacteria and strengthen the barrier function of the cervical epithelium, including anti-inflammatory effects (Branco et al., 2010; Huang et al., 2014; Anton et al., 2018; O’Hanlon et al., 2019). Under normal circumstances, this corresponds to the adaptive potential of healthy lactobacilli to natural disturbing factors (such as menses or intercourse) that affect their abundances (Kalra et al., 2007; Verstraelen et al., 2009; Santiago et al., 2012; Macklaim et al., 2011; Witkin et al., 2013; Beghini et al., 2015; Mayer et al., 2015; Abdelmaksoud et al., 2016; Lambert et al., 2013; Leizer et al., 2018; van der Veer et al., 2019). However, if these unfavorable conditions are long in duration, they can interfere with the resilience of lactobacilli to return to their original abundance. This may be an opportunity for better adaptable *L. iners* to establish itself the vaginal microbiota despite having the smallest genome among vaginal lactobacilli (~1.3 Mbp) and an insufficient ability to provide full nutrition (Macklaim et al., 2011). This adaptation can represent a selective advantage that enables *L. iners* to survive or even thrive in disturbed vaginal ecosystem and probably contributes to being the most widespread lactobacillus worldwide and why CST3 is the most common CST in women with dysbiotic or pathological microbiota (Kalra et al., 2007; Verstraelen et al., 2009; Petrova et al., 2017; Zheng et al., 2021). This is consistent with our results, in which CST3 was predominant in both controls and patients with CVD, and *L. iners* was the only microbe isolated from all samples processed during the study period in both cohorts. Interestingly, the significant difference in Nugent score (from 3 to 4) in CST3 during follow-up period had no relevant effect on CST3 transition (**Figure 4**). Furthermore, we found an antagonistic relationship between *L. iners* and *L. crispatus* regardless of clinical status, especially in the controls, yeast etiology, and CST1, that is, groups with an increased abundance of *L. crispatus*. Our results demonstrated that the *L. crispatus* load was closely related to CST1 regardless CVD etiology (**Figure 3A**) and this species can be an important part of CVD microbiota of the yeast etiology, which agrees with its presence in women with dysbiotic microbiota such as BV or VVC (Teixeira et al., 2012; Ravel et al., 2011). It seems that the role of *L. crispatus* (and other bacteria) in the vagina cannot be simplistically viewed from a one-sided perspective but must be considered in the context of interrelationships in the vaginal microbiota and environment, especially between lactobacilli and non-lactobacilli bacteria and/or yeasts, including their possible direct or indirect (e.g., through metabolic microbial nets) influences on microbial characteristics such as production of toxins, bacteriocins, D-lactate, and H_2_O_2_.

**Figure 6:**
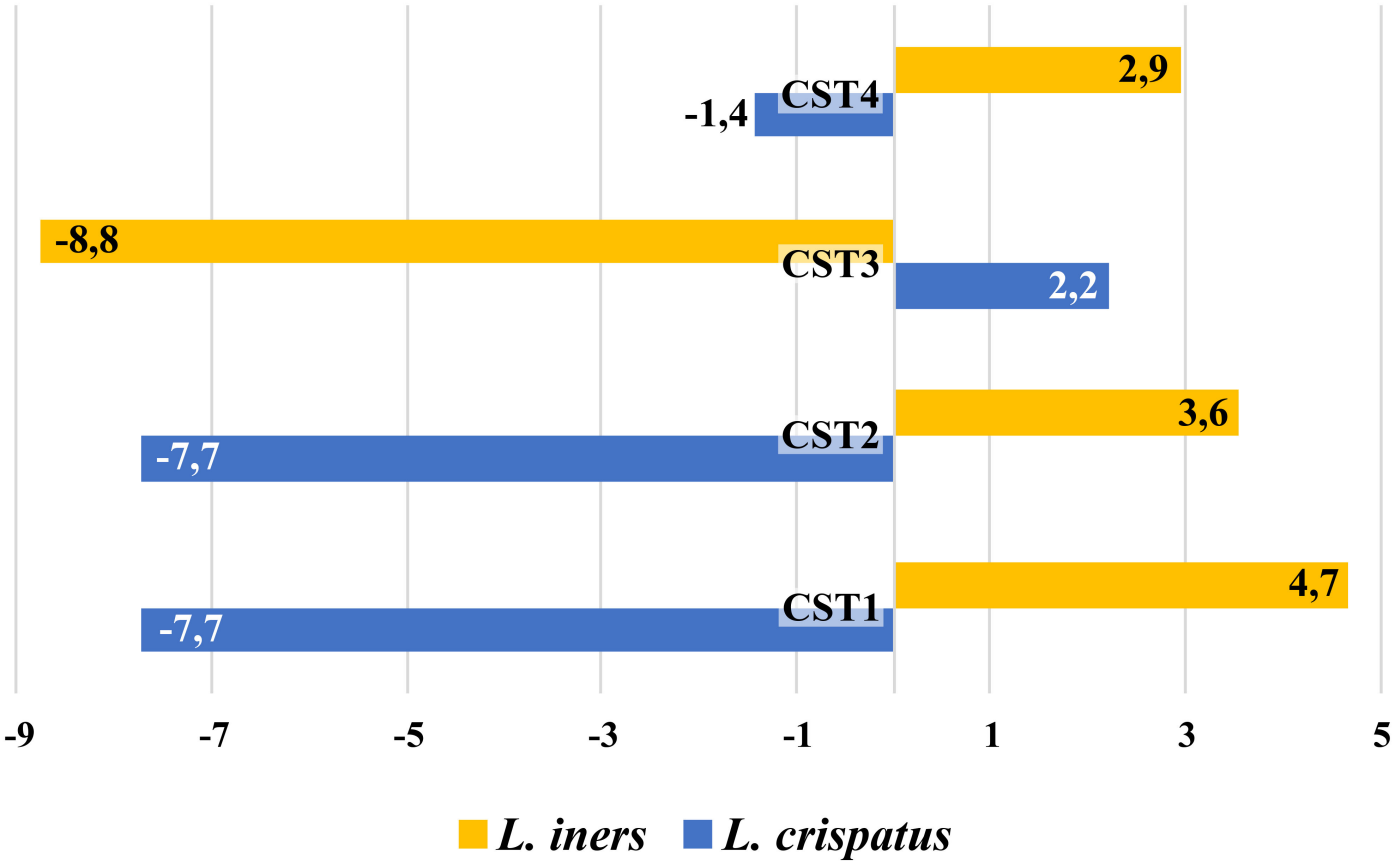
Comparison of cumulative correlation coefficient for *L. crispatus* and *L. iners* with other vaginal bacteria in CSTs.

These findings suggested that CST3/CST2 in CVD patients may be an alternative to a healthy microbiota, which is shaped by permanent pressure of unfavorable conditions associated with CVD and its management. One factor that could contribute to the selection of CST3 in CVD is metronidazole and/or other BV medications, as previously demonstrated by the results of topical treatment in patients with BV (Ferris et al., 2007; Jakobsson and Forsum, 2007; Brooks et al., 2017b). However, detailed analysis suggested that adaptation to CVD condition may be driven by a different mechanism in CST2 and CST3. While in CST3, resistance of *L. iners* to metronidazole seems to be the main selective mechanism, in case of *L. gasseri* (CST2), other selection mechanisms are likely at play. It was supported the fact that CST3 represented the only CST, in which *L. iners* took over the role of *L. crispatus* (in terms of interrelationships) as indicated by the positive ccc for *L. iners* with other bacteria in CST3 as well as *L. crispatus* in CST1, CST2, and CST4 (**Figure 6**). In our setting, long-term exposure of microbiota to adverse conditions of CVD led to a reduction of CST3/CST2 diversity (**Supplementary Figure S5**). This is a situation analogous to the hyperestrogenic status during pregnancy, which is associated with increased *L. crispatus* burden and lactic acid production accompanied by reduced diversity and relatively higher stability of the vaginal microbiota. Indeed, a similar effect was observed in women using hormonal contraception (van de Wijgert et al., 2013; Romero et al., 2014; Walther-António et al., 2014; DiGiulio et al., 2015; Zheng et al., 2019; Tuddenham et al., 2023). On the other hand, pregnancy and higher levels of female hormones can be viewed as a long-term stress factor that leads to similar consequences as the effect of chronic CVD conditions, as suggested by the high percentage of pregnant women colonized with *L. gasseri* and *L. iners*, which species have been associated with microbiota instability based on their reduced colonization strength and colonization resistance in the vagina (Verhelst et al., 2005; Verstraelen et al., 2009).

Our study highlights some methodological pitfalls in the evaluation and interpretation of the microbiota results. We are aware that species-level annotation focusing on 16S rRNA sequencing analysis is not optimal (Laudadio et al., 2018). Moreover, the detailed evaluation of our data at the level of CVD subgroups or individual CSTs was limited by the low number of women in some categories (especially CST5, CST2, unstable CSTs), which may have affected some results and their interpretation. Furthermore, taxonomic classification at the species level may appear inadequate for some vaginal bacteria that is, they may not capture the complex physiological connections and their consequences for the vaginal microbiome, as shown by studies suggesting functional differences within species (Ma et al., 2020; Holm et al., 2023). Another issue is the rather wide definition of CVD, and hence the relatively high heterogeneity of our CVD groups (specifically the mixed group), the classification of which was largely based on retrospective anamnestic data. Finally, there was also the possibility of sample bias; samples collected at the entry and control visits may not be representative of the dynamic nature of the vaginal microbiota, but only a snapshot with a time-limited distortion, making interpretation of the results difficult or misleading. This was particularly evident in women in the control group, with a relatively large degree of variability in the composition of the microbiota, which was most likely the result of fluctuations under the influence of natural external and internal factors to which the vaginal microbiome was exposed. On the other hand, long-term follow-up of two CVD patients with CST3 showed that the composition of the CST was relatively stable and did not change significantly, although in one patient, *L. jensenii* and *A. vaginae* alternated as the second most abundant species (**Supplementary Figure S8**). These conclusions need to be confirmed using larger data sets and long-term follow-up and therefore future studies should be designed as prospective with longitudinal sampling to obtain a sufficient number of patients in each (especially “rare”) category for deeper insight into the dynamics of the vaginal microbiota. To evaluate complex relationships in vaginal microbiome, use more sophisticated methods (metabolomics, etc.) is desirable. Since our study is focused on patients with chronic forms of CVD, it will be necessary to compare them with those who experienced an acute infection in order to understand the exact role of vaginal microbiota in individual etiologies of infectious origin.

## 5 Conclusion

Our results support the essential role of *Lactobacillus* species in the composition of the vaginal microbiota and shaping the vaginal environment. While changes in the abundance of lactobacilli are reflected substantially in the Nugent score, the physiological background represented by vaginal pH could be driven by not only lactobacilli but also alternative mechanisms. These findings suggest that bacterial vaginosis-type infections might be associated with the disbalance of vaginal microbiota as a result of *Lactobacillus* species-dependent effect of clinical status and stability of the CST. Our results revealed an opposite trend in abundance of *L. iners* and *L. gasseri* between CVD patients and healthy controls, depending on CST stability. CST shift in healthy controls lacked clinical manifestations and was indicated by an increase in *L. iners* abundance and a relative deficit of *L. gasseri* at entry visit. Overall, dysbiosis only became apparent at the control visit probably due to lower colonization resistance of *L. iners* and corresponded to a transient microbiota subsequently leading to a restoration of original state based on microbial resilience. In contrast, dysbiotic (labile) microbiota in CVD showed a decreased proportion of *L. iners* and an increased in *L. gasseri* at the entry visit, which coincided with CVD symptoms. During follow-up period, microbiota composition gradually approached a newly established microbial balance at the control visit accompanied by relief or resolution of signs and symptoms of a CVD episode, most likely because of therapeutic intervention. Due to recurrent episodes and (often inadequate) antibiotic therapy, persistent CVD distress acts as a selection factor for *L. iners* and CST3, thus maintaining the relative stability of CST3, as under normal circumstances a portion of CST3 would undergo CST shift. Thus, CST3 may serve as a resilience marker in CVD but as an instability marker in health. These results highlighted a different role and dynamics of index *Lactobacillus* species towards each other and towards non-lactobacilli depending on the clinical status and stability of CST and may contribute to the diagnosis of chronic forms of vaginal infections (bacterial or yeast origin). This increases the importance of clinical context and knowledge of the structure/stability of vaginal microbiota for the correct interpretation of the vaginal microbes/microbiota in both CVD and health conditions. If the clinical significance of these findings is confirmed, it could lead to a more effective diagnosis and management of CVD syndrome. Anyway, reconstruction or preservation of the natural microbiota should be a priority.

## Data Availability

The data presented in the study are deposited in the NCBI SRA repository, accession numbers PRJNA1301405 (part1) and PRJNA1301413 (part 2).

## References

[B1] Abdelmaksoud A. A. Koparde V. N. Sheth N. U. Serrano M. G. Glascock A. L. Fettweis J. M.. (2016). Comparison of *Lactobacillus crispatus* isolates from *Lactobacillus*-dominated vaginal microbiomes with isolates from microbiomes containing bacterial vaginosis-associated bacteria. Microbiology 162, 466–475. doi: 10.1099/mic.0.000238, PMID: 26747455 PMC4891990

[B2] Anton L. Sierra L. J. DeVine A. Barila G. Heiser L. Brown A. G.. (2018). Common cervicovaginal microbial supernatants alter cervical epithelial function: Mechanisms by which *Lactobacillus crispatus* contributes to cervical health. Front. Microbiol. 9. doi: 10.3389/fmicb.2018.02181, PMID: 30349508 PMC6186799

[B3] Antonio M. A. Hawes S. E. Hillier S. L. (1999). The identification of vaginal *Lactobacillus* species and the demographic and microbiologic characteristics of women colonized by these species. J. Infect. Dis. 180, 1950–1956. doi: 10.1086/315109, PMID: 10558952

[B4] Aroutcheva A. Ling Z. Faro S. (2008). *Prevotella bivia* as a source of lipopolysaccharide in the vagina. Anaerobe 14, 256–260. doi: 10.1016/j.anaerobe.2008.08.002, PMID: 18849004 PMC2651005

[B5] Beghini J. Linhares I. M. Giraldo P. C. Ledger W. J. Witkin S. S. (2015). Differential expression of lactic acid isomers, extracellular matrix metalloproteinase inducer, and matrix metalloproteinase-8 in vaginal fluid from women with vaginal disorders. BJOG 122, 1580–1585. doi: 10.1111/1471-0528.13072, PMID: 25196575

[B6] Bradshaw C. S. Tabrizi S. N. Fairley C. K. Morton A. N. Rudland E. Garland S. M. (2006). The association of *Atopobium vaginae* and *Gardnerella vaginalis* with bacterial vaginosis and recurrence after oral metronidazole therapy. J. Infect. Dis. 194, 828–836. doi: 10.1086/506621, PMID: 16941351

[B7] Branco K. M. Nardi R. M. Moreira J. L. Nunes A. C. Farias L. M. Nicoli J. R.. (2010). Identification and *in vitro* production of *Lactobacillus* antagonists from women with or without bacterial vaginosis. Braz. J. Med. Biol. Res. 43, 338–344. doi: 10.1590/s0100-879x2010007500013, PMID: 20209377

[B8] Brooks J. P. Buck G. A. Chen G. Diao L. Edwards D. J. Fettweis J. M.. (2017a). Changes in vaginal community state types reflect major shifts in the microbiome. Microb. Ecol. Health Dis. 28, 1303265. doi: 10.1080/16512235.2017.1303265, PMID: 28572753 PMC5443090

[B9] Brooks J. P. Edwards D. J. Blithe D. L. Fettweis J. M. Serrano M. G. Sheth N. U.. (2017b). Effects of combined oral contraceptives, depot medroxyprogesterone acetate and the levonorgestrel-releasing intrauterine system on the vaginal microbiome. Contraception 95, 405–413. doi: 10.1016/j.contraception.2016.11.006, PMID: 27913230 PMC5376524

[B10] Brown S. E. Schwartz J. A. Robinson C. K. O′Hanlon D. E. Bradford L. L. He X.. (2019). The vaginal microbiota and behavioral factors associated with genital *Candida albicans* detection in reproductive-age women. Sex Transm. Dis. 46, 753–758. doi: 10.1097/OLQ.0000000000001066, PMID: 31517769 PMC6818707

[B11] Callahan B. J. Sankaran K. Fukuyama J. A. McMurdie P. J. Holmes S. P. (2016). Bioconductor workflow for microbiome data analysis: from raw reads to community analyses. F1000Res 5, 1492. doi: 10.12688/f1000research.8986.2, PMID: 27508062 PMC4955027

[B12] Cole J. R. Wang Q. Fish J. A. Chai B. McGarrell D. M. Sun Y.. (2014). Ribosomal Database Project: data and tools for high throughput rRNA analysis. Nucleic Acids Res. 42, D633–D642. doi: 10.1093/nar/gkt1244, PMID: 24288368 PMC3965039

[B13] DiGiulio D. B. Callahan B. J. McMurdie P. J. Costello E. K. Lyell D. J. Robaczewska A.. (2015). Temporal and spatial variation of the human microbiota during pregnancy. Proc. Natl. Acad. Sci. U.S.A. 112, 11060–11065. doi: 10.1073/pnas.1502875112, PMID: 26283357 PMC4568272

[B14] Farage M. A. Miller K. W. Sobel J. D. (2010). Dynamics of the vaginal ecosystem—hormonal influences. Infect. Dis: Res. Treat. 3, 1–15. doi: 10.4137/IDRT.S3903

[B15] Ferris M. J. Norori J. Zozaya-Hinchliffe M. Martin D. H. (2007). Cultivation independent analysis of changes in bacterial vaginosis flora following metronidazole treatment. J. Clin. Microbiol. 45, 1016–1018. doi: 10.1128/JCM.02085-06, PMID: 17202272 PMC1829144

[B16] Fischer G. Bradford J. (2011). Persistent vaginitis. BMJ 28, 343:d7314. doi: 10.1136/bmj.d7314, PMID: 22127139

[B17] France M. Alizadeh M. Brown S. Ma B. Ravel J. (2022). Towards a deeper understanding of the vaginal microbiota. Nat. Microbiol. 7, 367–378. doi: 10.1038/s41564-022-01083-2, PMID: 35246662 PMC8910585

[B18] Gajer P. Brotman R. M. Bai G. Sakamoto J. Schütte U. M. Zhong X.. (2012). Temporal dynamics of the human vaginal microbiota. Sci. Transl. Med. 4, 132ra52. doi: 10.1126/scitranslmed.3003605, PMID: 22553250 PMC3722878

[B19] George S. D. Van Gerwen O. T. Dong C. Sousa L. G. V. Cerca N. Elnaggar J. H.. (2024). The role of *Prevotella* species in female genital tract infections. Pathogens 13, 364. doi: 10.3390/pathogens13050364, PMID: 38787215 PMC11123741

[B20] Holm J. B. France M. T. Gajer P. Ma B. Brotman R. M. Shardell M.. (2023). Integrating compositional and functional content to describe vaginal microbiomes in health and disease. Microbiome 11, 259. doi: 10.1186/s40168-023-01692-x, PMID: 38031142 PMC10688475

[B21] Huang B. Fettweis J. M. Brooks J. P. Jefferson K. K. Buck G. A. (2014). The changing landscape of the vaginal microbiome. Clin. Lab. Med. 34, 747–761. doi: 10.1016/j.cll.2014.08.006, PMID: 25439274 PMC4254509

[B22] Jakobsson T. Forsum U. (2007). *Lactobacillus iners*: a marker of changes in the vaginal flora? J. Clin. Microbiol. 45, 3145. doi: 10.1128/JCM.00558-07, PMID: 17652481 PMC2045263

[B23] Kaambo E. Africa C. Chambuso R. Passmore J. S. (2018). Vaginal microbiomes associated with aerobic vaginitis and bacterial vaginosis. Front. Public Health 6. doi: 10.3389/fpubh.2018.00078, PMID: 29632854 PMC5879096

[B24] Kalra A. Palcu C. T. Sobel J. D. Akins R. A. (2007). Bacterial vaginosis: culture- and PCR-based characterizations of a complex polymicrobial disease’s pathobiology. Curr. Infect. Dis. Rep. 9, 485–500. doi: 10.1007/s11908-007-0074-4, PMID: 17999885

[B25] Lambert J. A. John S. Sobel J. D. Akins R. A. (2013). Longitudinal analysis of vaginal microbiome dynamics in women with recurrent bacterial vaginosis: recognition of the conversion process. PloS One 8, e82599. doi: 10.1371/journal.pone.0082599, PMID: 24376552 PMC3869700

[B26] Laudadio I. Fulci V. Palone F. Stronati L. Cucchiara S. Carissimi C. (2018). Quantitative assessment of shotgun metagenomics and 16S rDNA amplicon sequencing in the study of human gut microbiome. OMICS 22, 248–254. doi: 10.1089/omi.2018.0013, PMID: 29652573

[B27] Lee C. Y. Dillard L. R. Papin J. A. Arnold K. B. (2023). New perspectives into the vaginal microbiome with systems biology. Trends Microbiol. 31, 356–368. doi: 10.1016/j.tim.2022.09.011, PMID: 36272885

[B28] Leizer J. Nasioudis D. Forney L. J. Schneider G. M. Gliniewicz K. Boester A.. (2018). Properties of epithelial cells and vaginal secretions in pregnant women when *Lactobacillus crispatus* or *Lactobacillus iners* dominate the vaginal microbiome. Reprod. Sci. 25, 854–860. doi: 10.1177/1933719117698583, PMID: 28301987

[B29] Lev-Sagie A. De Seta F. Verstraelen H. Ventolini G. Lonnee-Hoffmann R. Vieira-Baptista P. (2022). The vaginal microbiome: II. Vaginal dysbiotic conditions. J. Low Genit Tract Dis. 26, 79–84. doi: 10.1097/LGT.0000000000000644, PMID: 34928257 PMC8719518

[B30] Linhares I. M. Summers P. R. Larsen B. Giraldo P. C. Witkin S. S. (2011). Contemporary perspectives on vaginal pH and lactobacilli. Am. J. Obstet. Gynecol. 204, 120.e1–120.e5. doi: 10.1016/j.ajog.2010.07.010, PMID: 20832044

[B31] Ma B. Forney L. J. Ravel J. (2012). Vaginal microbiome: rethinking health and disease. Annu. Rev. Microbiol. 66, 371–389. doi: 10.1146/annurev-micro-092611-150157, PMID: 22746335 PMC3780402

[B32] Ma B. France M. T. Crabtree J. Holm J. B. Humphrys M. S. Brotman R. M.. (2020). A comprehensive non-redundant gene catalog reveals extensive within-community intraspecies diversity in the human vagina. Nat. Commun. 11, 940. doi: 10.1038/s41467-020-14677-3, PMID: 32103005 PMC7044274

[B33] MaChado A. Cerca N. (2015). Influence of biofilm formation by *Gardnerella vaginalis* and other anaerobes on bacterial vaginosis. J. Infect. Dis. 212, 1856–1861. doi: 10.1093/infdis/jiv338, PMID: 26080369

[B34] Macklaim J. M. Gloor G. B. Anukam K. C. Cribby S. Reid G. (2011). At the crossroads of vaginal health and disease, the genome sequence of *Lactobacillus iners* AB-1. Proc. Natl. Acad. Sc.i U.S.A. 108, 4688–4695. doi: 10.1073/pnas.1000086107, PMID: 21059957 PMC3063587

[B35] Martinez R. C. Franceschini S. A. Patta M. C. Quintana S. M. Nunes A. C. Moreira J. L.. (2008). Analysis of vaginal lactobacilli from healthy and infected Brazilian women. Appl. Environ. Microbiol. 74, 4539–4542. doi: 10.1128/AEM.00284-08, PMID: 18502927 PMC2493183

[B36] Mayer B. T. Srinivasan S. Fiedler T. L. Marrazzo J. M. Fredricks D. N. Schiffer J. T. (2015). Rapid and profound shifts in the vaginal microbiota following antibiotic treatment for bacterial vaginosis. J. Infect. Dis. 212, 793–802. doi: 10.1093/infdis/jiv079, PMID: 25676470 PMC4539900

[B37] McMurdie P. J. Holmes S. (2013). phyloseq: An R package for reproducible interactive analysis and graphics of microbiome census data. PloS One 8, e61217. doi: 10.1371/journal.pone.0061217, PMID: 23630581 PMC3632530

[B38] Novak J. Ravel J. Ma B. Ferreira C. S. T. Tristão A. D. R. Silva M. G (2022). Characteristics associated with *Lactobacillus iners*-dominated vaginal microbiota. Sex Transm Infect 98, 353–359. doi: 10.1136/sextrans-2020-054824, PMID: 34497114

[B39] Nugent R. P. Krohn M. A. Hillier S. L. (1991). Reliability of diagnosing bacterial vaginosis is improved by a standardized method of Gram stain interpretation. J. Clin. Microbiol. 29, 297–301. doi: 10.1128/jcm.29.2.297-301.1991, PMID: 1706728 PMC269757

[B40] Nyirjesy P. (2014). Management of persistent vaginitis. Obstet. Gynecol. 124, 1135–1146. doi: 10.1097/AOG.0000000000000551, PMID: 25415165

[B41] Nyirjesy P. Peyton C. Weitz M. V. Mathew L. Culhane J. F. (2006). Causes of chronic vaginitis: analysis of a prospective database of affected women. Obstet Gynecol. 108, 1185–1191. doi: 10.1097/01.AOG.0000239103.67452.1a, PMID: 17077241

[B42] O’Hanlon D. E. Come R. A. Moench T. R. (2019). Vaginal pH measured *in vivo*: lactobacilli determine pH and lactic acid concentration. BMC Microbiol. 19, 13. doi: 10.1186/s12866-019-1388-8, PMID: 30642259 PMC6332693

[B43] Pelayo P. Hussain F. A. Werlang C. A. Wu C. M. Woolston B. M. Xiang C. M.. (2024). *Prevotella* are major contributors of sialidases in the human vaginal microbiome. Proc. Natl. Acad. Sci. U.S.A. 121, e2400341121. doi: 10.1073/pnas.2400341121, PMID: 39186657 PMC11388281

[B44] Petrova M. I. Lievens E. Malik S. Imholz N. Lebeer S. (2015). *Lactobacillus* species as biomarkers and agents that can promote various aspects of vaginal health. Front. Physiol. 6. doi: 10.3389/fphys.2015.00081, PMID: 25859220 PMC4373506

[B45] Petrova M. I. Reid G. Vaneechoutte M. Lebeer S. (2017). *Lactobacillus iners*: friend or foe? Trends Microbiol. 25, 182–191. doi: 10.1016/j.tim.2016.11.007, PMID: 27914761

[B46] Plummer E. L. Sfameni A. M. Vodstrcil L. A. Danielewski J. A. Murray G. L. Fehler G.. (2023). *Prevotella* and *Gardnerella* are associated with treatment failure following first-line antibiotics for bacterial vaginosis. J. Infect. Dis. 228, 646–656. doi: 10.1093/infdis/jiad261, PMID: 37427495 PMC10469350

[B47] Pybus V. Onderdonk A. B. (1997). Evidence for a commensal, symbiotic relationship between *Gardnerella vaginalis* and *Prevotella bivia* involving ammonia: potential significance for bacterial vaginosis. J. Infect. Dis. 175, 406–413. doi: 10.1093/infdis/175.2.406, PMID: 9203662

[B48] Pybus V. Onderdonk A. B. (1998). A commensal symbiosis between *Prevotella bivia* and *Peptostreptococcus anaerobius* involves amino acids: potential significance to the pathogenesis of bacterial vaginosis. FEMS Immunol. Med. Microbiol. 22, 317–327. doi: 10.1111/j.1574-695X.1998.tb01221.x, PMID: 9879923

[B49] Quast C. Pruesse E. Yilmaz P. Gerken J. Schweer T. Yarza P.. (2013). The SILVA ribosomal RNA gene database project: improved data processing and web-based tools. Nucleic Acids Res. 41, D590–D596. doi: 10.1093/nar/gks1219, PMID: 23193283 PMC3531112

[B50] Ravel J. Brotman R. M. Gajer P. Ma B. Nandy M. Fadrosh D. W.. (2013). Daily temporal dynamics of vaginal microbiota before, during and after episodes of bacterial vaginosis. Microbiome 1, 29. doi: 10.1186/2049-2618-1-29, PMID: 24451163 PMC3968321

[B51] Ravel J. Gajer P. Abdo Z. Schneider G. M. Koenig S. S. McCulle S. L.. (2011). Vaginal microbiome of reproductive-age women. Proc. Natl. Acad. Sci. U.S.A. 108, 4680–4687. doi: 10.1073/pnas.1002611107, PMID: 20534435 PMC3063603

[B52] Romero R. Hassan S. S. Gajer P. Tarca A. L. Fadrosh D. W. Bieda J.. (2014). The vaginal microbiota of pregnant women who subsequently have spontaneous preterm labor and delivery and those with a normal delivery at term. Microbiome 2, 18. doi: 10.1186/2049-2618-2-18, PMID: 24987521 PMC4066267

[B53] Rönnqvist P. D. Forsgren-Brusk U. B. Grahn-Håkansson E. E. (2006). Lactobacilli in the female genital tract in relation to other genital microbes and vaginal pH. Acta Obstet. Gynecol. Scand. 85, 726–735. doi: 10.1080/00016340600578357, PMID: 16752267

[B54] Santiago G. L. Tency I. Verstraelen H. Verhelst R. Trog M. Temmerman M.. (2012). Longitudinal qPCR study of the dynamics of *L. crispatus*, *L. iners*, *A. vaginae*, (sialidase positive) *G. vaginalis*, and *P. bivia* in the vagina. PloS One 7, e45281. doi: 10.1371/journal.pone.0045281, PMID: 23028904 PMC3448655

[B55] Schellenberg J. J. Patterson M. H. Hill J. E. (2017). *Gardnerella vaginalis* diversity and ecology in relation to vaginal symptoms. Res. Microbiol. 168, 837–844. doi: 10.1016/j.resmic.2017.02.011, PMID: 28341009

[B56] Segata N. Izard J. Waldron L. Gevers D. Miropolsky L. Garrett W. S.. (2011). Metagenomic biomarker discovery and explanation. Genome Biol. 12, R60. doi: 10.1186/gb-2011-12-6-r60, PMID: 21702898 PMC3218848

[B57] Si J. You H. J. Yu J. Sung J. Ko G. (2017). *Prevotella* as a hub for vaginal microbiota under the influence of host genetics and their association with obesity. Cell Host Microbe 21, 97–105. doi: 10.1016/j.chom.2016.11.010, PMID: 28017660

[B58] Sobel J. D. Vempati Y. S. (2024). Bacterial vaginosis and vulvovaginal candidiasis pathophysiologic interrelationship. Microorganisms 12, 108. doi: 10.3390/microorganisms12010108, PMID: 38257934 PMC10820109

[B59] Song J. Dong X. Lan Y. Lu Y. Liu X. Kang X.. (2024). Interpretation of vaginal metagenomic characteristics in different types of vaginitis. mSystems 9, e0137723. doi: 10.1128/msystems.01377-23, PMID: 38364107 PMC10949516

[B60] Sousa L. G. V. Novak J. França A. Muzny C. A. Cerca N. (2024). *Gardnerella vaginalis*, *Fannyhessea vaginae*, and *Prevotella bivia* strongly influence each other’s transcriptome in triple-species biofilms. Microb. Ecol. 87, 117. doi: 10.1007/s00248-024-02433-9, PMID: 39294302 PMC11410844

[B61] Teixeira G. S. Carvalho F. P. Arantes R. M. E. Nunes A. C. Moreira J. L. S. Mendonça M.. (2012). Characteristics of *Lactobacillus* and *Gardnerella vaginalis* from women with or without bacterial vaginosis and their relationships in gnotobiotic mice. J. Med. Microbiol. 61, 1074–1081. doi: 10.1099/jmm.0.041962-0, PMID: 22539000

[B62] Tuddenham S. Gajer P. Burke A. E. Murphy C. Klein S. L. Stennett C. A.. (2023). *Lactobacillus*-dominance and rapid stabilization of vaginal microbiota in combined oral contraceptive pill users examined through a longitudinal cohort study with frequent vaginal sampling over two years. EBioMedicine 87, 104407. doi: 10.1016/j.ebiom.2022.104407, PMID: 36529102 PMC9792759

[B63] van der Veer C. Hertzberger R. Y. Bruisten S. M. Tytgat H. L. P. Swanenburg J. de Kat Angelino-Bart A.. (2019). Comparative genomics of human *Lactobacillus crispatus* isolates reveals genes for glycosylation and glycogen degradation: implications for *in vivo* dominance of the vaginal microbiota. Microbiome 7, 49. doi: 10.1186/s40168-019-0667-9, PMID: 30925932 PMC6441167

[B64] van de Wijgert J. H. Borgdorff H. Verhelst R. Crucitti T. Francis S. Verstraelen H.. (2014). The vaginal microbiota: what have we learned after a decade of molecular characterization? PloS One 9, e105998. doi: 10.1371/journal.pone.0105998, PMID: 25148517 PMC4141851

[B65] van de Wijgert J. H. Jespers V. (2017). The global health impact of vaginal dysbiosis. Res. Microbiol. 168, 859e864. doi: 10.1016/j.resmic.2017.02.003, PMID: 28257809

[B66] van de Wijgert J. H. Verwijs M. C. Turner A. N. Morrison C. S. (2013). Hormonal contraception decreases bacterial vaginosis but oral contraception may increase candidiasis: implications for HIV transmission. AIDS 27, 2141–2153. doi: 10.1097/QAD.0b013e32836290b6, PMID: 23660575

[B67] Ventolini G. Vieira-Baptista P. De Seta F. Verstraelen H. Lonnee-Hoffmann R. Lev-Sagie A. (2022). The Vaginal Microbiome: IV. The Role of vaginal microbiome in reproduction and in gynecologic cancers. J. Low Genit Tract Dis. 26, 93–98. doi: 10.1097/LGT.0000000000000646, PMID: 34928259 PMC8719507

[B68] Verhelst R. Verstraelen H. Claeys G. Verschraegen G. Van Simaey L. De Ganck C.. (2005). Comparison between Gram stain and culture for the characterization of vaginal microflora: definition of a distinct grade that resembles grade I microflora and revised categorization of grade I microflora. BMC Microbiol. 5, 61. doi: 10.1186/1471-2180-5-61, PMID: 16225680 PMC1266370

[B69] Verstraelen H. Verhelst R. Claeys G. De Backer E. Temmerman M. Vaneechoutte M. (2009). Longitudinal analysis of the vaginal microflora in pregnancy suggests that *L. crispatus* promotes the stability of the normal vaginal microflora and that *L. gasseri* and/or *L. iners* are more conducive to the occurrence of abnormal vaginal microflora. BMC Microbiol. 9, 116. doi: 10.1186/1471-2180-9-116, PMID: 19490622 PMC2698831

[B70] Verstraelen H. Vieira-Baptista P. De Seta F. Ventolini G. Lonnee-Hoffmann R. Lev-Sagie A. (2022). The vaginal microbiome: I. Research development, lexicon, defining “Normal” and the dynamics throughout women’s lives. J. Low Genit Tract Dis. 26, 73–78. doi: 10.1097/LGT.0000000000000643, PMID: 34928256 PMC8719517

[B71] Walther-António M. R. Jeraldo P. Berg Miller M. E. Yeoman C. J. Nelson K. E. Wilson B. A.. (2014). Pregnancy’s stronghold on the vaginal microbiome. PloS One 9, e98514. doi: 10.1371/journal.pone.0098514, PMID: 24896831 PMC4045671

[B72] Weinstein M. M. Prem A. Jin M. Tang S. Bhasin J. M. (2019). FIGARO: An efficient and objective tool for optimizing microbiome rRNA gene trimming parameters. bioRxiv, 610394. doi: 10.1101/610394

[B73] Witkin S. S. Mendes-Soares H. Linhares I. M. Jayaram A. Ledger W. J. Forney L. J. (2013). Influence of vaginal bacteria and D- and L-lactic acid isomers on vaginal extracellular matrix metalloproteinase inducer: implications for protection against upper genital tract infections. MBio 4, e00460-13. doi: 10.1128/mBio.00460-13, PMID: 23919998 PMC3735189

[B74] Zheng N. Guo R. Wang J. Zhou W. Lin Z. (2021). Contribution of *Lactobacillus iners* to vaginal health and diseases: A Systematic Review. Front. Cell. Infect. Microbiol. 11. doi: 10.3389/fcimb.2021.792787, PMID: 34881196 PMC8645935

[B75] Zheng N. Guo R. Yao Y. Jin M. Cheng Y. Ling Z. (2019). *Lactobacillus iners* is associated with vaginal dysbiosis in healthy pregnant women: A preliminary study. BioMed. Res. Int. 2019, 6079734. doi: 10.1155/2019/6079734, PMID: 31781627 PMC6855029

