# Supplementary material for: Vaginal microbiota: different roles of lactobacilli and community instability in chronic vulvovaginal discomfort
Source: Front Cell Infect Microbiol. 2025 Aug 18;15:1636873. doi: 10.3389/fcimb.2025.1636873 (PMC12399642; doi:10.3389/fcimb.2025.1636873)
Supplement: Supplementary file 1 [file DataSheet1.docx]

Supplementary Material

# Supplementary Data

## Supplementary Figures and Tables

Figure S1 Distribution of CSTs in healthy controls and CVD patients at entry (In) and control (Out) visit

Figure S2 Comparison of composition of CST4 subtypes by clinical status (CVD vs. health)

Figure S3 Distribution of unstable and stable CSTs in CST categories by clinical status (CVD versus healthy controls).

Figure S4 Comparison of α-diversity of stable and unstable CSTs in vaginal microbiota of healthy controls (A) and CVD group (B)

Figure S5 Comparison of α-diversity of vaginal microbiota in CSTs and CVD etiology groups

Figure S6 Comparison of cumulative correlation coefficient (ccc) of *Lactobacillus* and non-*Lactobacillus* species in unstable and stable CSTs of CVD patients and healthy controls

Figure S7 Comparison of cumulative correlation coefficient (ccc) of *Lactobacillus* and non-*Lactobacillus* species in unstable and stable CSTs within CVD groups (A) and CST categories (B)

Figure S8 Long-term changes in composition of vaginal microbiota of CST3 in two patients with CVD

Table S1 Comparison of vaginal microbiota in healthy controls, CVD group, and CVD etiologies

Table S2 Comparison of vaginal microbiota of unstable and stable CSTs in CVD group and healthy controls (Ctrl)

Table S3 Correlations between all bacterial taxa tested in CVD patients and the control group at entry (In) and control (Out) visit

## Supplementary Figures and Tables

**Figure S1** Distribution of CSTs in healthy controls and CVD patients at entry (In) and control (Out) visit. Note: Figures in columns represent number of CSTs. In/Out - entry/control visit

**Figure S2** Comparison of composition of CST4 subtypes by clinical status (CVD vs. health). CST4 subtypes: BV-like - *Atopobium*, *Megasphaera*, *Sneathia*; G+ cocci - all except of *S. agalactiae*; SRAG - *Streptococcus agalactiae*, Str-o - streptococci other than SRAG, Ente - *Enterococcus*, AECH -*Aerococcus christensenii*, Staph - *Staphylococcus* spp., LACR - *Lactobacillus crispatus*, LAGA - *L. gasseri*, LAIN - *L. iners*, LARH - *L. rhamnosus*, Lact sp - *Lactobacillus* spp. except for LACR, LAIN, LAGA, and LARH, Mega - *Megasphaera* spp., Veillo - *Veillonella* spp., GAVA - *Gardnerella* *vaginalis*, ATVA - *Atopobium vaginae*, Bifido - Bifidobacteriales, PRBI - *Prevotella bivia*, Pre-o - *Prevotella* spp. other than PRBI, SNAM - *Sneathia amnii*, Others - species negligibly represented, NA - unidentified species

**Figure S3** Distribution of unstable and stable CSTs in CST categories by clinical status (CVD versus healthy controls). Note: Figures in columns represent number of CSTs. In/Out - entry/control visit


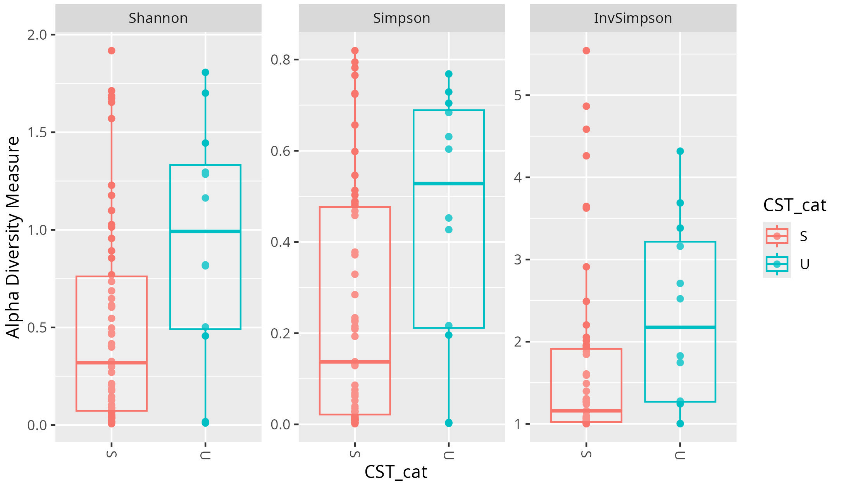

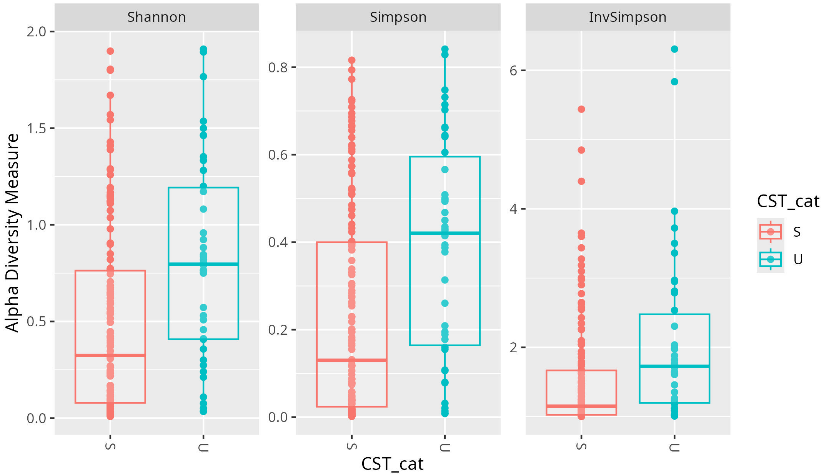
A B

**Figure S4** Comparison of α-diversity of stable and unstable CSTs in vaginal microbiota of healthy controls (A) and CVD group (B). S/U - stable/unstable CSTs, CST_cat - CST category


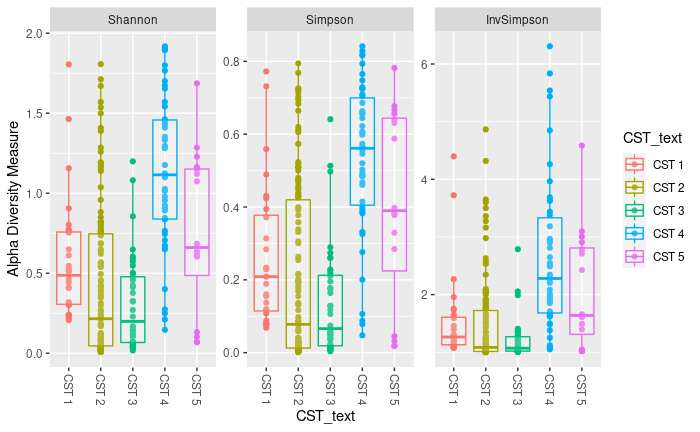


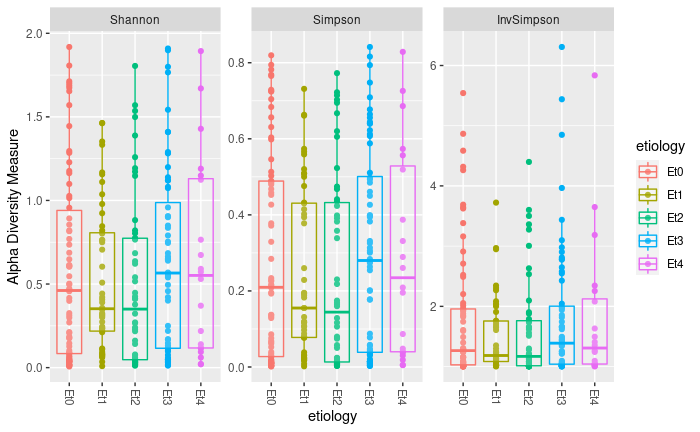


**Figure S5** Comparison of α-diversity of vaginal microbiota in CSTs and CVD etiology groups.

Et0 - control group, Et1 - non-specific etiology, Et2 - yeast etiology, Et3 - bacterial etiology, Et4 - mixed etiology


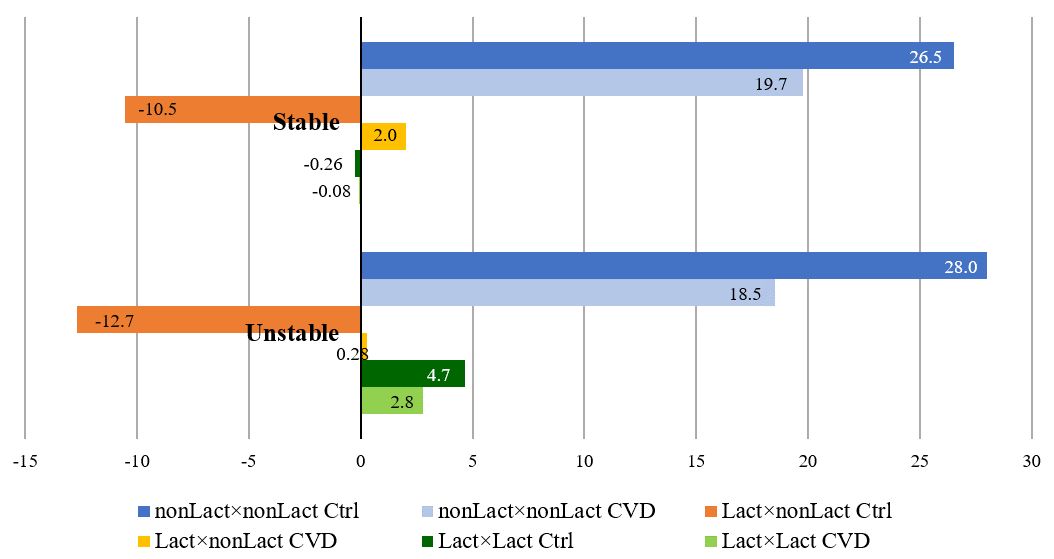


**Figure S6** Comparison of cumulative correlation coefficient (ccc) of *Lactobacillus* and non-*Lactobacillus* species in unstable and stable CSTs of CVD patients and healthy controls (Ctrl). Lact - *Lactobacillus* spp., nonLact - non-*Lactobacillus* spp.

*Lactobacillus* × *Lactobacillus* *Lactobacillus* × non-*Lactobacillus* non-*Lactobacillus* × non-*Lactobacillus*

**Figure S7** Comparison of cumulative correlation coefficient (ccc) of *Lactobacillus* and non-*Lactobacillus* species in unstable and stable CSTs within CVD groups and CST categories. Ctrl - control group, Et1 - non-specific etiology, Et2 - yeast etiology, Et3 - bacterial etiology, Et4 - mixed etiology


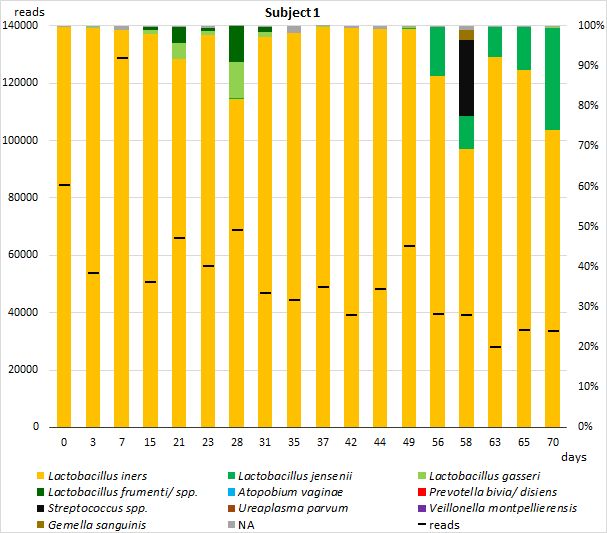


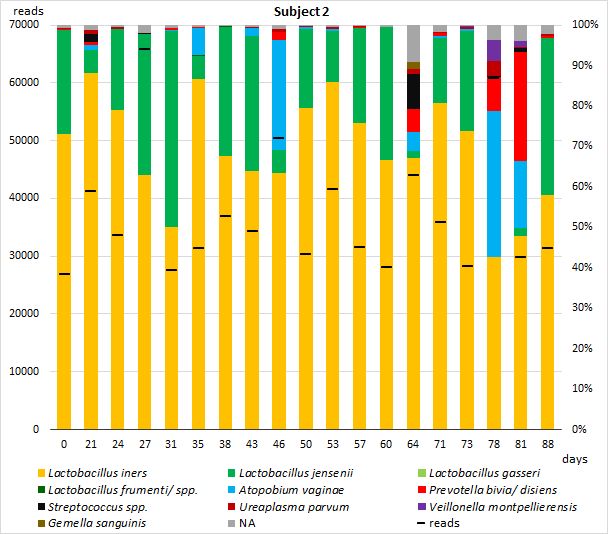


**Figure S8** Long-term changes in composition of vaginal microbiota of CST3 in two patients with CVD. axis x – number of days, axis y - relative abundance (%, right), black line – a number of reads (corresponding with axis y, left), NA - not identified

**Table S1** Comparison of vaginal microbiota in healthy controls (Ctrl), CVD group, and CVD etiologies.

Et1 - non-specific etiology, Et2 - yeast etiology, Et3 - bacterial etiology, Et4 - mixed etiology

Firm – Firmicutes, Actino – Actinomycetes, Bacter – Bacteroidetes, Fuso – Fusobacteria, Proteo – Proteobacteria, Teneri – Tenericutes, NA - other bacteria (not identified), Lact-all – *Lactobacillus* spp., G+cocci – G-positive cocci, Prev-all – *Prevotella* spp., LACR - *Lactobacillus crispatus*, LAIN - *L. iners*, LAGA - *L. gasseri*, LAJE - *L. jensenii*, Lact sp - *Lactobacillus* spp. except for LACR, LAIN, LAGA, and LAJE, SRAG - *Streptococcus agalactiae*, Str-o - streptococci other than SRAG, Ente - *Enterococcus* spp., AECH -*Aerococcus christensenii*, Staph - *Staphylococcus* spp., Clostr - Clostridiales, Mega - *Megasphaera* spp., Veillo - *Veillonella* spp., Dialis - *Dialister* spp., Acti-o - other Actinomycetales, Bifi - Bifidobacteriales, GAVA - *Gardnerella vaginalis*, ATVA - *Atopobium* *vaginae*, Bact-o - other Bacteroidales, PRBI - *Prevotella bivia*, Pre-o - *Prevotella* spp. other than PRBI, SNAM - *Sneathia amnii*, FUNU - *Fusobacterium nucleatum*

BV-like = G+cocci, Clostr, Veillo, Mega, ATVA, GAVA, Bifi, Prev-all, FUNU, SNAM

bounded - statistically significant also by parametric test (t‑test or paired samples t‑test)

|  | **Ctrl** | **CVD** | **Et1** | **Et2** | **Et3** | **Et4** | **Ctrl** |  | **CVD** |  | P^a^ | P^b^ | P^b^ | P ^a^ | P ^a^ | P ^a^ | P ^a^ | P ^a^ | P ^a^ | P ^a^ | P ^a^ | P ^a^ | P ^a^ |
| --- | --- | --- | --- | --- | --- | --- | --- | --- | --- | --- | --- | --- | --- | --- | --- | --- | --- | --- | --- | --- | --- | --- | --- |
| Median (%) | All | All | All | All | All | All | In | Out | In | Out | ctrl×cvd | ctrl×ctrl | cvd×cvd | Ctrl×Et1 | Ctrl×Et2 | Ctrl×Et3 | Ctrl×Et4 | Et1×Et2 | Et1×Et3 | Et1×Et4 | Et2×Et3 | Et2×Et4 | Et3×Et4 |
| n | 70 | 182 | 48 | 50 | 64 | 20 | 35 | 35 | 91 | 91 | All×All | In×Out | In×Out | All×All | All×All | All×All | All×All | All×All | All×All | All×All | All×All | All×All | All×All |
| Firm | 99.24 | 98.00 | 97.95 | 99.00 | 97.66 | 96.10 | 99.28 | 99.16 | 97.97 | 98.03 | 0.1642 | 0.1025 | 0.1746 | 0.2096 | 0.8480 | 0.0957 | 0.1350 | 0.1205 | 0.7913 | 0.3601 | 0.0522 | 0.0467 | 0.5082 |
| Actino | 0.055 | 0.017 | 0.034 | 0.006 | 0.018 | 0.013 | 0.019 | 0.071 | 0.015 | 0.017 | 0.0675 | 0.7141 | 0.4060 | 0.1798 | 0.0199 | 0.4908 | 0.2870 | 0.3025 | 0.4059 | 0.7148 | 0.1073 | 0.6732 | 0.4999 |
| Bacter | 0.033 | 0.030 | 0.064 | 0.019 | 0.035 | 0.026 | 0.020 | 0.043 | 0.032 | 0.028 | 0.6773 | 0.2028 | 0.1572 | 0.3280 | 0.1020 | 0.9378 | 0.4997 | 0.0179 | 0.3542 | 0.3286 | 0.1219 | 0.7196 | 0.6546 |
| Fuso | 0.001 | 0 | 0.003 | 0 | 0.0005 | 0 | 0 | 0.001 | 0 | 0 | 0.8650 | 0.1477 | 0.8248 | 0.1327 | 0.0259 | 0.5355 | 0.2991 | 0.0009 | 0.5090 | 0.0882 | 0.0135 | 0.6650 | 0.1903 |
| Proteo | 0 | 0 | 0 | 0 | 0 | 0 | 0 | 0 | 0 | 0 | 0.1118 | 0.0830 | 0.6322 | 0.3205 | 0.1695 | 0.0795 | 0.7337 | 0.7165 | 0.5506 | 0.2750 | 0.8453 | 0.1376 | 0.0761 |
| Teneri | 0 | 0 | 0 | 0 | 0 | 0 | 0 | 0 | 0 | 0 | 0.7170 | 0.7422 | 0.7002 | 0.0374 | 0.3140 | 0.4181 | 0.3408 | 0.0082 | 0.1727 | 0.0089 | 0.1079 | 0.8781 | 0.1424 |
| NA | 0.357 | 1.168 | 1.242 | 0.698 | 1.239 | 1.818 | 0.337 | 0.38 | 1.2 | 1.136 | 0.0002 | 0.7432 | 0.8308 | 0.0017 | 0.0711 | 0.0013 | 0.0029 | 0.1098 | 0.6423 | 0.2786 | 0.1510 | 0.0245 | 0.1593 |
| Lact-all | 98.53 | 97.76 | 97.48 | 98.83 | 95.57 | 95.03 | 99.19 | 96.21 | 97.38 | 97.84 | 0.1784 | 0.0797 | 0.2103 | 0.2511 | 0.4823 | 0.0314 | 0.1836 | 0.0528 | 0.3762 | 0.5903 | 0.0042 | 0.0460 | 0.7848 |
| G+cocci | 0.030 | 0.015 | 0.047 | 0.002 | 0.017 | 0.016 | 0.029 | 0.031 | 0.015 | 0.018 | 0.0840 | 0.6643 | 0.2373 | 0.4221 | <0.0001 | 0.6160 | 0.6035 | <0.0001 | 0.2842 | 0.3323 | 0.0006 | 0.0075 | 0.9329 |
| Prev-all | 0.025 | 0.028 | 0.064 | 0.012 | 0.031 | 0.026 | 0.020 | 0.039 | 0.028 | 0.027 | 0.7108 | 0.2110 | 0.1564 | 0.2791 | 0.0960 | 0.9485 | 0.5439 | 0.0150 | 0.3391 | 0.3738 | 0.1275 | 0.6152 | 0.6853 |
| BV-like | 0.134 | 0.141 | 0.018 | 0.141 | 0.155 | 0.436 | 0.114 | 0.637 | 0.149 | 0.137 | 0.9623 | 0.2482 | 0.4826 | 0.0011 | 0.1097 | 0.8065 | 0.7709 | 0.0456 | 0.0012 | 0.0076 | 0.3026 | 0.1250 | 0.5494 |
| LACR | 0.319 | 0.152 | 0.497 | 0.137 | 0.152 | 0.034 | 0.366 | 0.272 | 0.174 | 0.141 | 0.1624 | 0.7247 | 0.3904 | 0.6434 | 0.3247 | 0.1934 | 0.1147 | 0.6390 | 0.4606 | 0.1805 | 0.9476 | 0.2863 | 0.2960 |
| LAIN | 8.39 | 38.03 | 1.35 | 62.26 | 4.64 | 25.43 | 19.75 | 7.13 | 12.26 | 57.55 | 0.6371 | 0.1085 | 0.4520 | 0.7778 | 0.1068 | 0.9947 | 0.9574 | 0.0678 | 0.8141 | 0.9839 | 0.1259 | 0.2168 | 0.8418 |
| LAGA | 0.021 | 0.037 | 0.099 | 0.033 | 0.013 | 0.015 | 0.021 | 0.035 | 0.050 | 0.034 | 0.7282 | 0.3926 | 0.8747 | 0.0005 | 0.8292 | 0.1372 | 0.4315 | 0.0031 | <0.0001 | 0.0061 | 0.3893 | 0.6527 | 1 |
| LAJE | 0.017 | 0.027 | 0.045 | 0.039 | 0.020 | 0.007 | 0.018 | 0.014 | 0.027 | 0.027 | 0.5835 | 0.5496 | 0.6743 | 0.1566 | 0.3168 | 0.8673 | 0.0521 | 0.8422 | 0.2103 | 0.0127 | 0.3650 | 0.0417 | 0.0854 |
| Lact sp | 0.050 | 0.026 | 0.026 | 0.008 | 0.043 | 0.056 | 0.028 | 0.053 | 0.027 | 0.018 | 0.6948 | 0.7309 | 0.7864 | 0.6690 | 0.3190 | 0.9236 | 0.6941 | 0.5379 | 0.7060 | 0.6370 | 0.4143 | 0.3543 | 0.7479 |
| SRAG | 0 | 0 | 0 | 0 | 0 | 0 | 0 | 0 | 0 | 0 | 0.1100 | 0.4258 | 0.2278 | 0.0025 | 0.1994 | 0.1134 | 0.3328 | 0.0002 | 0.1739 | 0.2101 | 0.0122 | 0.0518 | 0.8144 |
| Strep sp | 0.003 | 0.002 | 0.009 | 0 | 0.002 | 0.003 | 0.003 | 0.002 | 0.002 | 0.002 | 0.6668 | 0.5926 | 0.6262 | 0.1393 | 0.0173 | 0.7405 | 0.8541 | 0.0003 | 0.0733 | 0.3162 | 0.0385 | 0.0366 | 0.6029 |
| Entero | 0 | 0 | 0 | 0 | 0 | 0 | 0 | 0 | 0 | 0 | 0.9017 | 0.2163 | 0.5609 | 0.0629 | 0.3244 | 0.7473 | 0.0351 | 0.0076 | 0.1339 | 0.0012 | 0.2098 | 0.1262 | 0.0258 |
| AECH | 0 | 0 | 0 | 0 | 0 | 0 | 0 | 0 | 0 | 0 | 0.7828 | 0.0250 | 0.0224 | 0.4741 | 0.0570 | 0.9517 | 0.5307 | 0.0066 | 0.4848 | 0.9211 | 0.0665 | 0.0514 | 0.5049 |
| Staph | 0.006 | 0.001 | 0.003 | 0 | 0.0005 | 0.003 | 0.006 | 0.005 | 0.001 | 0.001 | 0.0002 | 0.4624 | 0.8128 | 0.4143 | <0.0001 | 0.0018 | 0.2203 | 0.0002 | 0.0424 | 0.6553 | 0.0381 | 0.0211 | 0.3706 |
| Clostr | 0.003 | 0.001 | 0 | 0 | 0.004 | 0.022 | 0.002 | 0.004 | 0.001 | 0.001 | 0.0335 | 0.2343 | 0.6507 | 0.0022 | 0.0207 | 0.4537 | 0.5306 | 0.5616 | 0.0168 | 0.0060 | 0.0971 | 0.0279 | 0.2611 |
| Mega | 0 | 0 | 0 | 0 | 0 | 0 | 0 | 0 | 0 | 0 | 0.4102 | 0.8906 | 0.4366 | 0.7206 | 0.0921 | 0.9577 | 0.0918 | 0.0564 | 0.8396 | 0.0673 | 0.1516 | 0.5718 | 0.1086 |
| Veillo | 0 | 0 | 0.001 | 0 | 0 | 0 | 0 | 0.001 | 0 | 0 | 0.9778 | 0.0605 | 0.1152 | 0.4879 | 0.5121 | 0.5708 | 0.1314 | 0.1715 | 0.8006 | 0.0538 | 0.2547 | 0.3422 | 0.0726 |
| Dialis | 0 | 0 | 0 | 0 | 0 | 0.017 | 0 | 0 | 0 | 0 | 0.3551 | 0.0432 | 0.3102 | 0.0057 | 0.5554 | 0.5997 | 0.0314 | 0.0198 | 0.0114 | 0.0004 | 0.8834 | 0.0347 | 0.0242 |
| GAVA | 0 | 0 | 0 | 0 | 0 | 0 | 0 | 0 | 0 | 0 | 0.0003 | 0.7381 | 0.3072 | 0.0059 | 0.0005 | 0.0090 | 0.7499 | 0.5147 | 0.6468 | 0.0631 | 0.2404 | 0.0124 | 0.1232 |
| ATVA | 0 | 0 | 0 | 0 | 0 | 0 | 0 | 0 | 0 | 0 | 0.0171 | 0.7344 | 0.0295 | 0.2656 | 0.0076 | 0.1783 | 0.2190 | 0.1232 | 0.9348 | 0.6738 | 0.1162 | 0.3323 | 0.6812 |
| Actino-o | 0.002 | 0 | 0 | 0 | 0.001 | 0.0005 | 0.002 | 0.002 | 0 | 0 | 0.1387 | 0.1658 | 0.9647 | 0.1247 | 0.1722 | 0.3747 | 0.8366 | 0.8517 | 0.4938 | 0.3470 | 0.5666 | 0.4181 | 0.7375 |
| Bifido-o | 0.003 | 0 | 0.002 | 0 | 0.002 | 0 | 0.002 | 0.003 | 0 | 0 | 0.0074 | 0.1280 | 0.5675 | 0.3298 | 0.0032 | 0.4426 | <0.0001 | 0.0851 | 0.7231 | 0.0003 | 0.0739 | 0.0093 | 0.0005 |
| PRBI | 0 | 0 | 0 | 0 | 0 | 0 | 0 | 0 | 0 | 0 | 0.0009 | 0.3225 | 0.8408 | 0.0690 | 0.0347 | 0.0214 | 0.0016 | 0.7378 | 0.8742 | 0.0289 | 0.8879 | 0.0442 | 0.0301 |
| Prev sp | 0.010 | 0.014 | 0.024 | 0.008 | 0.012 | 0.024 | 0.009 | 0.010 | 0.014 | 0.012 | 0.4328 | 0.8141 | 0.1878 | 0.0711 | 0.8185 | 0.6778 | 0.7963 | 0.0414 | 0.1591 | 0.6320 | 0.5744 | 0.5789 | 0.9579 |
| Bacter-o | 0.007 | 0.004 | 0.012 | 0.0005 | 0.006 | 0.002 | 0.004 | 0.017 | 0.005 | 0.003 | 0.1053 | 0.2557 | 0.2026 | 0.9978 | 0.0051 | 0.3310 | 0.3549 | 0.0073 | 0.3866 | 0.4953 | 0.0771 | 0.4613 | 0.7133 |
| SNAM | 0 | 0 | 0.0005 | 0 | 0 | 0 | 0 | 0 | 0 | 0 | 0.3759 | 0.0728 | 0.5955 | 0.3671 | 0.0101 | 0.6513 | 0.0060 | 0.0012 | 0.7420 | 0.0015 | 0.0063 | 0.1962 | 0.0049 |
| FUNU | 0 | 0 | 0 | 0 | 0 | 0 | 0 | 0 | 0 | 0 | 0.8342 | 0.6257 | 0.8980 | 0.4413 | 0.3462 | 0.5848 | 0.7330 | 0.1135 | 0.2145 | 0.8214 | 0.6754 | 0.3194 | 0.4962 |

P^a^ – Mann-Whitney test, P^b^ - Wilcoxon test (paired samples)

**Table S2** Comparison of vaginal microbiota of unstable and stable CSTs in CVD group and healthy controls (Ctrl).

Stab - stable CSTs, UnSt –unstable CSTs (labile + new CSTs), StA/StB = stable CSTs at entry (A)/control visit (B), P^a^ – Mann-Whitney test, P^b^ - Wilcoxon test (paired samples). Abbreviations: see Table S1

| Median | **CVD** | **Ctrl** | P ^a^ | **CVD** | **Ctrl** | P | **Ctrl** | **Ctrl** | **Ctrl** | **Ctrl** | **CVD** | **CVD** | **CVD** | **CVD** | P ^a^ | P ^a^ | P ^a^ | P ^a^ | P ^a^ | P ^a^ | P ^a^ | P ^a^ | P ^b^ | P ^b^ | P ^b^ | P ^b^ |
| --- | --- | --- | --- | --- | --- | --- | --- | --- | --- | --- | --- | --- | --- | --- | --- | --- | --- | --- | --- | --- | --- | --- | --- | --- | --- | --- |
| (%) | Stab | Stab | Cvd×Ctrl | UnStab | UnStab | cvd×ctrl | Lab | StA | New | StB | Lab | StA | New | StB | Ctrl×Cvd | Ctrl×Cvd | Ctrl×Cvd | Ctrl×Cvd | Ctrl×Ctrl | Ctrl×Ctrl | Cvd×Cvd | Cvd×Cvd | Ctrl×Ctrl | Ctrl×Ctrl | Cvd×Cvd | Cvd×Cvd |
|  | All | All | Stab×Stab | All | All | UnSt×UnSt | In | In | Out | Out | In | In | Out | Out | Lab×Lab | New×New | StA×StA | StB×StB | Lab×StA | New×StB | Lab×StA | New×StB | Lab×New | StA×StB | Lab×New | StA×StB |
| n | 140 | 58 | 140×58 | 42 | 12 | 42×12 | 6 | 29 | 6 | 29 | 21 | 70 | 21 | 70 | In×In | Out×Out | In×In | Out×Out | In×In | Out×Out | In×In | Out×Out | In×Out | In×Out | In×Out | In×Out |
| Firm | 98.65 | 99.44 | 0.1494 | 93.40 | 94.81 | 0.9502 | 97.33 | 99.38 | 63.95 | 99.47 | 86.56 | 98.91 | 96.07 | 98.55 | 0.0706 | 0.0620 | 0.2186 | 0.4374 | 0.8610 | 0.0286 | 0.0001 | 0.0535 | 0.1563 | 0,1219 | 0,2074 | 0,7855 |
| Actino | 0.015 | 0.016 | 0.1553 | 0.126 | 3.228 | 0.0937 | 1.43 | 0.019 | 23.26 | 0.013 | 1.33 | 0.0135 | 0.02 | 0.017 | 0.9535 | 0.0227 | 0.2745 | 0.3558 | 0.4053 | 0.0254 | 0.0094 | 0.3935 | 0.0938 | 0,2891 | 0,2614 | 0,7967 |
| Bacter | 0.021 | 0.021 | 0.3121 | 0.130 | 0.186 | 0.6773 | 0.019 | 0.020 | 0.540 | 0.022 | 0.238 | 0.021 | 0.088 | 0.022 | 0.0662 | 0.1615 | 0.5743 | 0.4057 | 0.7759 | 0.0597 | 0.0007 | 0.0311 | 0.2188 | 0,1396 | 0,3813 | 0,5583 |
| Fuso | 0 | 0 | 0.4029 | 0.016 | 0.003 | 0.4143 | 0.0005 | 0 | 0.023 | 0.001 | 0.017 | 0 | 0.004 | 0 | 0.0374 | 0.3416 | 0.4350 | 0.7053 | 0.8297 | 0.0783 | 0.0004 | 0.0414 | 0.0625 | 0,2979 | 0,9005 | 0,4552 |
| Proteo | 0 | 0 | 0.0205 | 0 | 0 | 0.3283 | 0 | 0 | 0 | 0 | 0 | 0 | 0 | 0 | 0.3362 | 0.6024 | 0.0266 | 0.3238 | 0.1240 | 0.9427 | 0.8039 | 0.0415 |  | 0,4375 | 0,3880 | 0,9658 |
| Teneri | 0 | 0 | 0.8780 | 0 | 0 | 0.6750 | 0 | 0 | 0 | 0 | 0 | 0 | 0 | 0 | 0.5930 | 0.3688 | 0.6891 | 0.5295 | 0.4173 | 0.8939 | 0.3400 | 0.4029 |  |  | 0,3293 | 1,0000 |
| NA | 0.906 | 0.333 | 0.0008 | 3.08 | 1.51 | 0.1511 | 0.7575 | 0.337 | 2.67 | 0.326 | 3.82 | 0.8455 | 2.65 | 1.00 | 0.0474 | 0.7706 | 0.0245 | 0.0159 | 0.9651 | 0.0256 | <0.0001 | 0.0936 | 0.4375 | 0,1592 | 0,4198 | 0,2624 |
| Lact-all | 98.55 | 99.23 | 0.2375 | 81.64 | 90.91 | 0.8352 | 96.84 | 99.36 | 37.34 | 99.15 | 48.42 | 98.75 | 87.55 | 98.42 | 0.0908 | 0.1025 | 0.2749 | 0.5851 | 0.7928 | 0.0320 | 0.0001 | 0.0662 | 0.1563 | 0,1219 | 0,0760 | 0,8652 |
| G+cocci | 0.010 | 0.019 | 0.0133 | 0.122 | 0.035 | 0.5738 | 0.010 | 0.042 | 0.167 | 0.015 | 0.065 | 0.005 | 0.257 | 0.014 | 0.0797 | 0.5595 | 0.0097 | 0.3993 | 0.2549 | 0.0877 | 0.0026 | 0.1696 | 0.4375 | 0,5949 | 0,2791 | 0,0523 |
| Prev-all | 0.018 | 0.021 | 0.3174 | 0.130 | 0.169 | 0.6102 | 0.018 | 0.020 | 0.484 | 0.022 | 0.238 | 0.0145 | 0.088 | 0.021 | 0.0705 | 0.2207 | 0.5664 | 0.4145 | 0.7927 | 0.0487 | 0.0006 | 0.0304 | 0.2188 | 0,1396 | 0,3816 | 0,5563 |
| BV-like | 0.070 | 0.110 | 0.6628 | 4.41 | 6.24 | 0.8760 | 0.106 | 0.114 | 47.14 | 0.097 | 22.252 | 0.071 | 1.58 | 0.068 | 0.0473 | 0.1025 | 0.4722 | 0.9877 | 0.9825 | 0.0203 | 0.0006 | 0.0479 | 0.1563 | 0,0386 | 0,0261 | 0,5243 |
| LACR | 0.142 | 0.319 | 0.1230 | 0.170 | 0.547 | 0.9502 | 10.66 | 0.366 | 0.526 | 0.272 | 0.174 | 0.164 | 0.169 | 0.124 | 0.9535 | 0.9767 | 0.2682 | 0.2649 | 0.9129 | 0.7098 | 0.6889 | 0.7883 | 0.5625 | 0,7151 | 0,4609 | 0,4160 |
| LAIN | 52.55 | 6.01 | 0.4406 | 2.43 | 21.31 | 0.3491 | 71.20 | 6.83 | 10.42 | 3.87 | 0.855 | 48.374 | 46.16 | 59.99 | 0.0358 | 0.4142 | 0.6501 | 0.5060 | 0.6616 | 0.7928 | 0.0221 | 0.6785 | 0.0938 | 0,0537 | 0,0065 | 0,7321 |
| LAGA | 0.027 | 0.024 | 0.4808 | 0.133 | 0.014 | 0.0469 | 0.014 | 0.021 | 0.021 | 0.041 | 0.246 | 0.033 | 0.083 | 0.025 | 0.1152 | 0.2934 | 0.8565 | 0.4145 | 0.7097 | 0.5992 | 0.0061 | 0.0852 | 0.6875 | 0,8894 | 0,9926 | 0,7865 |
| LAJE | 0.031 | 0.017 | 0.4469 | 0.024 | 0.012 | 0.9336 | 0.011 | 0.020 | 0.013 | 0.014 | 0.027 | 0.029 | 0.016 | 0.031 | 0.8610 | 1.0000 | 0.9081 | 0.3229 | 0.4567 | 0.7424 | 0.2540 | 0.1010 | 0.8438 | 0,6894 | 0,3503 | 0,7619 |
| Lact sp | 0.027 | 0.052 | 0.7381 | 0.021 | 0.008 | 0.8758 | 0.006 | 0.089 | 0.067 | 0.051 | 0.026 | 0.033 | 0.013 | 0.026 | 0.3347 | 0.5201 | 0.6552 | 0.9203 | 0.2638 | 0.5690 | 0.8910 | 0.5552 | 0.1563 | 0,5412 | 0,2971 | 0,9057 |
| SRAG | 0 | 0 | 0.8553 | 0 | 0 | 0.0100 | 0 | 0 | 0 | 0 | 0 | 0 | 0 | 0 | 0.1119 | 0.0435 | 0.8808 | 0.6919 | 0.2806 | 0.2302 | 0.0675 | 0.0365 |  | 0,3394 | 0,8825 | 0,5217 |
| Strep sp | 0.0015 | 0.003 | 0.1780 | 0.016 | 0.0025 | 0.1972 | 0.002 | 0.003 | 0.003 | 0.001 | 0.03 | 0.002 | 0.008 | 0.001 | 0.1577 | 0.7195 | 0.1947 | 0.5590 | 0.5589 | 0.8396 | 0.0008 | 0.1140 | 0.6875 | 0,0665 | 0,3058 | 0,2821 |
| Entero | 0 | 0 | 0.8053 | 0 | 0 | 0.6698 | 0 | 0 | 0 | 0 | 0 | 0 | 0.001 | 0 | 0.9424 | 0.6149 | 0.9023 | 0.8178 | 0.7540 | 0.6213 | 0.4367 | 0.0221 |  | 0,7910 | 0,3312 | 0,3458 |
| AECH | 0 | 0 | 0.8286 | 0 | 0 | 0.2195 | 0 | 0 | 0.052 | 0 | 0 | 0 | 0 | 0 | 0.8727 | 0.0630 | 0.9077 | 0.8640 | 0.7661 | 0.0618 | 0.9410 | 0.9689 | 0.1250 | 0,3125 | 0,2925 | 0,0417 |
| Staph | 0.001 | 0.005 | 0.0003 | 0.0015 | 0.017 | 0.1090 | 0.008 | 0.006 | 0.119 | 0.004 | 0.011 | 0 | 0 | 0.001 | 0.8340 | 0.0259 | 0.0024 | 0.0414 | 0.8433 | 0.0332 | 0.1117 | 0.7478 | 0.1250 | 0,2734 | 0,3210 | 0,5004 |
| Clostr | 0.0005 | 0.002 | 0.0733 | 0.003 | 0.043 | 0.1363 | 0.008 | 0.002 | 0.545 | 0.003 | 0.008 | 0.0005 | 0.001 | 0.0005 | 0.9047 | 0.0374 | 0.4516 | 0.0761 | 0.7342 | 0.1119 | 0.1919 | 0.5205 | 0.0625 | 0,5245 | 0,4237 | 0,9280 |
| Mega | 0 | 0 | 0.0427 | 0.002 | 0 | 0.2574 | 0 | 0 | 0 | 0 | 0.002 | 0 | 0.002 | 0 | 0.5296 | 0.2753 | 0.2043 | 0.1090 | 0.8361 | 0.4332 | 0.0058 | 0.0114 |  | 0,3652 | 0,0964 | 0,7971 |
| Veillo | 0 | 0 | 0.7018 | 0.003 | 0.004 | 0.9404 | 0 | 0 | 0.064 | 0 | 0.004 | 0 | 0.001 | 0 | 0.0791 | 0.0571 | 0.7992 | 0.4340 | 0.9794 | 0.0199 | 0.0009 | 0.0948 | 0.0625 | 0,0546 | 0,1442 | 0,6714 |
| Dialis | 0 | 0 | 0.5218 | 0 | 0.006 | 0.3862 | 0.004 | 0 | 0.025 | 0 | 0 | 0 | 0 | 0 | 0.9478 | 0.1998 | 0.8081 | 0.4997 | 0.4956 | 0.1743 | 0.3128 | 0.6231 | 0.3125 | 0,1289 | 0,7487 | 0,7989 |
| GAVA | 0 | 0 | 0.0017 | 0 | 0.0005 | 0.0424 | 0.0005 | 0 | 0.012 | 0 | 0 | 0 | 0 | 0 | 0.2949 | 0.0754 | 0.0038 | 0.1599 | 0.9063 | 0.2560 | 0.4781 | 0.8527 | 0.2500 | 0,0625 | 0,2068 | 0,8620 |
| ATVA | 0 | 0 | 0.0253 | 0 | 0 | 0.3545 | 0 | 0 | 0 | 0 | 0 | 0 | 0 | 0 | 0.3895 | 0.7084 | 0.5424 | 0.0079 | 0.4155 | 0.5923 | 0.7977 | 0.5254 |  | 0,3750 | 0,3426 | 0,0488 |
| Actino-o | 0 | 0.001 | 0.5803 | 0 | 0.197 | 0.0591 | 0.126 | 0.001 | 0.279 | 0.001 | 0 | 0.001 | 0 | 0 | 0.3073 | 0.0949 | 0.7082 | 0.6667 | 0.2395 | 0.0590 | 0.7239 | 0.9836 | 0.5625 | 0,7910 | 0,4553 | 0,9020 |
| Bifido-o | 0 | 0.002 | 0.0044 | 0.004 | 0.401 | 0.2557 | 0.004 | 0.002 | 13.35 | 0.003 | 0.004 | 0 | 0.002 | 0 | 0.9047 | 0.0909 | 0.0244 | 0.0743 | 0.7227 | 0.1817 | 0.0441 | 0.1570 | 0.1250 | 0,1514 | 0,0647 | 0,7713 |
| PRBI | 0 | 0 | 0.0434 | 0 | 0.001 | 0.0005 | 0.0005 | 0 | 0.007 | 0 | 0 | 0 | 0 | 0 | 0.0314 | 0.0064 | 0.3276 | 0.0681 | 0.4098 | 0.1453 | 0.3215 | 0.5104 |  | 1,0000 | 0,3673 | 0,9399 |
| Prev sp | 0.010 | 0.007 | 0.7069 | 0.052 | 0.045 | 0.7312 | 0.016 | 0.009 | 0.150 | 0.007 | 0.057 | 0.0105 | 0.052 | 0.008 | 0.0702 | 0.1993 | 0.9079 | 0.6936 | 0.8778 | 0.0085 | 0.0045 | 0.0309 | 0.3125 | 0,1327 | 0,0545 | 0,5586 |
| Bact-o | 0.002 | 0.007 | 0.0293 | 0.045 | 0.121 | 0.9584 | 0.003 | 0.006 | 0.237 | 0.007 | 0.086 | 0.002 | 0.016 | 0.002 | 0.1971 | 0.1440 | 0.3301 | 0.0353 | 0.9645 | 0.1244 | 0.0007 | 0.0305 | 0.3125 | 0,1815 | 0,7525 | 0,8423 |
| SNAM | 0 | 0 | 0.3338 | 0 | 0.002 | 0.6807 | 0.0005 | 0 | 0.023 | 0 | 0.002 | 0 | 0 | 0 | 0.4033 | 0.1668 | 0.8216 | 0.2620 | 0.6323 | 0.1040 | 0.0193 | 0.1892 | 0.1250 | 0,4697 | 0,4106 | 1,0000 |
| FUNU | 0 | 0 | 0.4279 | 0 | 0 | 0.5561 | 0 | 0 | 0.0005 | 0 | 0 | 0 | 0 | 0 | 0.3119 | 0.8726 | 0.2324 | 0.9271 | 0.4589 | 0.1017 | 0.0649 | 0.0224 |  | 0,4238 | 0,3797 | 0,2297 |

**Table S3** Correlations between all bacterial taxa tested in CVD patients and the control group at entry (In) and control (Out) visit.

| **CVD** | **In** |  |  |  |  |  |  |  |  |  |  |  |  |  |  |  |  |  |  |  |  |  |  |
| --- | --- | --- | --- | --- | --- | --- | --- | --- | --- | --- | --- | --- | --- | --- | --- | --- | --- | --- | --- | --- | --- | --- | --- |
| **Out** | LACR | LAIN | LAGA | LAJE | Lact sp | SRAG | Str_o | Ente | AECH | Staph | Clostr | Mega | Veillo | Dialis | GAVA | ATVA | Acti_o | Bifi_o | PRBI | Prev_o | Bact_o | SNAM | FUNU |
| LACR |  | **-0.345** | **0.284** | 0.258 | 0.248 | -0.074 | 0.048 | 0.031 | -0.210 | 0.021 | **-0.314** | -0.026 | -0.048 | **-0.291** | **-0.337** | -0.160 | -0.151 | 0.009 | -0.266 | -0.123 | -0.083 | 0.053 | 0.111 |
| LAIN | **-0.499** |  | **-0.290** | -0.133 | -0.122 | -0.122 | **-0.357** | 0.024 | 0.019 | **-0.433** | -0.126 | -0.008 | -0.216 | -0.021 | -0.104 | -0.194 | -0.118 | -0.072 | 0.190 | -0.228 | -0.149 | -0.068 | -0.034 |
| LAGA | **0.358** | -0.185 |  | 0.183 | 0.134 | 0.254 | **0.338** | 0.214 | -0.055 | **0.320** | -0.210 | -0.083 | 0.194 | -0.167 | -0.114 | 0.141 | 0.051 | 0.164 | -0.090 | 0.137 | 0.071 | 0.063 | 0.182 |
| LAJE | 0.175 | -0.197 | 0.056 |  | 0.119 | 0.035 | 0.040 | 0.054 | -0.048 | 0.018 | **-0.269** | -0.187 | -0.005 | -0.181 | -0.069 | -0.088 | -0.068 | 0.041 | 0.011 | 0.015 | -0.119 | -0.111 | -0.066 |
| Lact sp | 0.214 | -0.156 | 0.203 | 0.196 |  | 0.156 | 0.200 | 0.156 | 0.100 | 0.186 | -0.012 | 0.055 | 0.122 | 0.097 | -0.067 | -0.010 | 0.108 | -0.016 | 0.027 | 0.257 | 0.117 | -0.083 | 0.107 |
| SRAG | 0.061 | -0.115 | 0.104 | 0.020 | 0.091 |  | **0.328** | 0.045 | -0.047 | **0.388** | -0.069 | -0.158 | -0.018 | -0.084 | 0.123 | -0.008 | -0.130 | 0.057 | -0.033 | 0.041 | 0.012 | 0.031 | 0.058 |
| Strep sp | 0.129 | **-0.365** | 0.112 | 0.018 | **0.326** | **0.292** |  | 0.246 | 0.057 | **0.494** | 0.071 | 0.090 | **0.561** | -0.010 | 0.220 | **0.331** | 0.020 | 0.086 | 0.187 | **0.359** | **0.369** | 0.231 | **0.361** |
| Entero | 0.135 | -0.070 | **0.304** | 0.054 | 0.122 | 0.163 | **0.299** |  | 0.251 | **0.272** | -0.046 | **0.278** | 0.218 | -0.167 | -0.042 | 0.258 | -0.165 | 0.256 | 0.157 | 0.238 | 0.140 | 0.233 | 0.103 |
| AECH | 0.059 | -0.067 | 0.223 | 0.034 | 0.124 | -0.032 | -0.061 | 0.184 |  | 0.189 | 0.163 | 0.263 | 0.230 | 0.110 | 0.131 | -0.078 | 0.214 | **0.278** | 0.098 | **0.300** | **0.324** | **0.386** | -0.129 |
| Staph | 0.236 | **-0.415** | 0.201 | **0.280** | 0.147 | **0.491** | **0.405** | **0.338** | 0.166 |  | -0.006 | -0.165 | **0.277** | -0.157 | **0.285** | **0.324** | 0.031 | 0.125 | -0.037 | 0.200 | 0.154 | 0.076 | 0.109 |
| Clostr | -0.211 | -0.074 | -0.244 | -0.151 | -0.051 | -0.028 | 0.099 | -0.144 | 0.028 | -0.080 |  | 0.178 | **0.310** | **0.502** | 0.154 | 0.177 | 0.181 | 0.087 | 0.013 | **0.565** | **0.492** | 0.101 | 0.053 |
| Mega | 0.105 | 0.078 | 0.157 | -0.031 | 0.075 | 0.178 | 0.058 | **0.441** | 0.267 | -0.013 | -0.051 |  | 0.149 | 0.017 | -0.084 | -0.115 | 0.111 | **0.386** | 0.111 | **0.373** | **0.504** | **0.545** | 0.065 |
| Veillo | 0.111 | -0.018 | -0.086 | -0.057 | 0.092 | 0.059 | **0.342** | **0.376** | -0.029 | 0.143 | 0.163 | 0.226 |  | 0.139 | 0.125 | 0.238 | 0.147 | 0.217 | **0.301** | **0.445** | **0.513** | **0.361** | **0.443** |
| Dialis | -0.266 | 0.050 | -0.264 | -0.155 | 0.013 | -0.104 | 0.071 | -0.224 | -0.132 | -0.150 | **0.669** | -0.111 | 0.062 |  | 0.179 | 0.039 | **0.447** | 0.018 | 0.161 | **0.359** | 0.222 | -0.169 | 0.063 |
| GAVA | -0.159 | -0.227 | -0.222 | 0.104 | -0.090 | 0.161 | 0.131 | 0.048 | -0.035 | **0.337** | 0.157 | -0.067 | 0.008 | 0.240 |  | 0.163 | 0.068 | -0.152 | 0.200 | 0.036 | -0.019 | -0.029 | 0.065 |
| ATVA | 0.011 | -0.113 | 0.059 | -0.093 | -0.006 | -0.043 | 0.235 | 0.232 | -0.106 | 0.078 | 0.003 | 0.111 | 0.087 | -0.012 | 0.104 |  | -0.058 | 0.062 | 0.261 | 0.202 | 0.088 | -0.071 | 0.163 |
| Actino-o | 0.022 | -0.125 | 0.098 | 0.130 | **0.328** | -0.174 | 0.110 | 0.031 | 0.242 | -0.021 | 0.028 | 0.240 | 0.090 | **0.284** | 0.106 | -0.121 |  | 0.251 | 0.213 | 0.240 | 0.123 | 0.097 | -0.066 |
| Bifido-o | 0.131 | -0.057 | **0.374** | 0.153 | 0.082 | 0.006 | -0.104 | **0.319** | **0.399** | 0.093 | -0.137 | **0.384** | 0.146 | -0.127 | -0.147 | -0.016 | **0.401** |  | 0.205 | **0.393** | 0.368 | **0.478** | 0.024 |
| PRBI | -0.231 | 0.174 | -0.233 | -0.119 | 0.051 | -0.021 | -0.006 | 0.147 | 0.036 | -0.034 | -0.005 | 0.138 | **0.342** | 0.168 | 0.175 | -0.009 | 0.246 | 0.161 |  | 0.036 | 0.024 | 0.201 | 0.154 |
| Prev sp | -0.126 | -0.075 | 0.057 | 0.004 | -0.073 | 0.197 | 0.187 | **0.369** | 0.234 | **0.273** | **0.349** | 0.242 | **0.289** | **0.286** | 0.095 | 0.069 | 0.199 | 0.212 | 0.150 |  | **0.678** | **0.289** | 0.156 |
| Bacter-o | 0.067 | -0.079 | 0.232 | -0.055 | 0.066 | 0.072 | 0.159 | **0.296** | **0.367** | 0.061 | **0.311** | **0.465** | **0.312** | 0.193 | -0.138 | -0.064 | **0.313** | **0.387** | 0.021 | **0.482** |  | **0.429** | **0.274** |
| SNAM | 0.096 | -0.041 | **0.273** | -0.029 | -0.052 | 0.087 | -0.001 | **0.415** | **0.396** | 0.095 | -0.036 | **0.517** | 0.221 | **-0.301** | -0.218 | 0.030 | 0.046 | **0.550** | -0.090 | **0.286** | **0.411** |  | 0.064 |
| FUNU | 0.094 | 0.009 | 0.093 | -0.046 | 0.022 | -0.049 | **0.354** | 0.230 | -0.037 | -0.017 | 0.159 | 0.157 | **0.325** | 0.183 | -0.015 | 0.073 | 0.111 | -0.020 | 0.018 | **0.275** | **0.272** | 0.043 |  |

bolded = p<0.01

| **Ctrl** | **In** |  |  |  |  |  |  |  |  |  |  |  |  |  |  |  |  |  |  |  |  |  |  |
| --- | --- | --- | --- | --- | --- | --- | --- | --- | --- | --- | --- | --- | --- | --- | --- | --- | --- | --- | --- | --- | --- | --- | --- |
| **Out** | LACR | LAIN | LAGA | LAJE | Lact sp | SRAG | Str_o | Ente | AECH | Staph | Clostr | Mega | Veillo | Dialis | GAVA | ATVA | Acti_o | Bifi_o | PRBI | Prev_o | Bact_o | SNAM | FUNU |
| LACR |  | **-0.730** | **0.406** | 0.287 | 0.097 | 0.067 | 0.206 | -0.155 | -0.240 | 0.214 | **-0.031** | -0.153 | 0.108 | **-0.225** | **0.189** | 0.188 | -0.374 | -0.335 | -0.142 | 0.001 | 0.092 | 0.023 | -0.106 |
| LAIN | **-0.503** |  | **-0.297** | -0.162 | -0.270 | -0.194 | **-0.474** | 0.145 | 0.022 | **-0.368** | -0.232 | -0.106 | -0.248 | -0.184 | -0.354 | -0.339 | 0.074 | 0.096 | 0.149 | -0.292 | -0.373 | -0.238 | -0.248 |
| LAGA | **0.562** | -0.137 |  | 0.243 | -0.058 | -0.005 | **0.281** | -0.179 | 0.063 | **0.061** | 0.199 | 0.058 | 0.224 | 0.223 | -0.021 | 0.094 | 0.063 | -0.202 | 0.043 | 0.062 | 0.365 | -0.111 | -0.247 |
| LAJE | 0.503 | -0.019 | 0.497 |  | 0.117 | -0.245 | 0.132 | 0.287 | -0.225 | 0.089 | **-0.018** | -0.494 | 0.016 | -0.311 | 0.035 | -0.277 | -0.342 | -0.300 | -0.115 | 0.060 | -0.034 | -0.158 | -0.180 |
| Lact sp | 0.197 | -0.199 | 0.078 | 0.250 |  | 0.241 | 0.371 | 0.055 | -0.185 | 0.494 | 0.165 | 0.053 | 0.273 | 0.181 | 0.361 | 0.080 | 0.034 | -0.079 | 0.116 | 0.165 | 0.147 | -0.236 | 0.343 |
| SRAG | -0.176 | 0.082 | -0.170 | -0.340 | 0.121 |  | **0.170** | -0.236 | -0.069 | **0.221** | -0.060 | 0.346 | -0.172 | 0.003 | 0.023 | 0.014 | 0.141 | -0.022 | -0.269 | -0.113 | -0.116 | 0.104 | -0.020 |
| Strep sp | -0.139 | **-0.392** | 0.057 | -0.154 | **0.172** | **0.349** |  | -0.257 | 0.066 | **0.442** | 0.545 | -0.051 | **0.513** | 0.520 | 0.546 | **0.438** | 0.023 | -0.045 | 0.139 | **0.513** | **0.444** | -0.078 | **0.305** |
| Entero | 0.086 | 0.080 | **0.229** | 0.168 | 0.049 | 0.065 | **0.001** |  | 0.166 | **0.014** | 0.051 | **0.006** | -0.167 | -0.072 | 0.076 | -0.138 | 0.067 | -0.010 | 0.276 | 0.171 | 0.114 | 0.066 | -0.188 |
| AECHi | -0.165 | 0.041 | -0.206 | -0.303 | -0.075 | -0.041 | -0.053 | 0.254 |  | 0.094 | 0.254 | 0.194 | 0.167 | 0.319 | -0.032 | 0.092 | 0.278 | **0.391** | -0.077 | **0.425** | **0.385** | **0.450** | 0.046 |
| Staph | -0.092 | **-0.137** | -0.199 | **0.080** | 0.372 | **-0.004** | **0.137** | **0.167** | 0.222 |  | 0.312 | -0.068 | **0.311** | 0.290 | **0.552** | **0.229** | 0.297 | 0.087 | 0.096 | 0.208 | 0.326 | 0.120 | 0.383 |
| Clostr | -0.176 | -0.360 | -0.241 | -0.348 | 0.092 | 0.113 | 0.553 | -0.059 | 0.065 | 0.303 |  | 0.210 | **0.701** | **0.649** | 0.323 | 0.394 | 0.174 | 0.216 | 0.269 | **0.746** | **0.605** | 0.073 | 0.230 |
| Mega | -0.070 | 0.014 | -0.150 | -0.276 | -0.429 | 0.032 | -0.049 | **0.071** | 0.272 | -0.252 | -0.089 |  | 0.062 | 0.296 | -0.082 | -0.042 | 0.415 | **0.374** | -0.027 | **0.010** | **0.359** | **0.212** | 0.184 |
| Veillo | -0.005 | -0.519 | -0.136 | -0.240 | 0.261 | -0.006 | **0.495** | **-0.136** | 0.052 | 0.381 | 0.593 | -0.090 |  | 0.508 | 0.469 | 0.373 | 0.182 | 0.264 | **0.228** | **0.547** | **0.554** | **-0.058** | **0.330** |
| *Dialister* | -0.285 | -0.329 | -0.201 | -0.508 | -0.003 | 0.024 | 0.476 | -0.072 | 0.304 | 0.186 | **0.597** | 0.041 | 0.350 |  | 0.266 | 0.477 | **0.438** | 0.317 | 0.429 | **0.580** | 0.494 | -0.022 | 0.281 |
| GAVA | 0.167 | -0.060 | 0.049 | 0.128 | 0.451 | -0.079 | 0.018 | 0.501 | -0.081 | **0.359** | 0.225 | -0.222 | 0.189 | 0.051 |  | 0.493 | 0.226 | 0.131 | 0.138 | 0.234 | 0.319 | -0.137 | 0.344 |
| ATVA | -0.083 | -0.509 | -0.178 | -0.250 | 0.265 | -0.049 | 0.364 | -0.145 | -0.147 | 0.032 | 0.442 | -0.151 | 0.530 | 0.439 | 0.196 |  | 0.000 | 0.120 | 0.203 | 0.362 | 0.118 | -0.090 | 0.049 |
| Actino-o | -0.409 | -0.040 | -0.148 | -0.229 | **-0.007** | 0.032 | 0.119 | 0.120 | 0.288 | 0.398 | 0.334 | 0.076 | 0.264 | **0.388** | 0.255 | 0.103 |  | 0.510 | 0.162 | 0.002 | 0.411 | 0.206 | 0.203 |
| Bifido-o | -0.294 | -0.100 | **-0.557** | -0.563 | -0.108 | -0.012 | 0.059 | **-0.056** | **0.487** | 0.090 | 0.339 | **0.350** | 0.341 | 0.529 | -0.063 | 0.212 | **0.264** |  | 0.120 | **0.163** | 0.275 | **0.338** | 0.413 |
| PRBI | -0.119 | 0.048 | -0.097 | -0.034 | 0.333 | 0.212 | 0.113 | 0.253 | -0.068 | 0.252 | 0.431 | -0.277 | **0.139** | 0.130 | 0.656 | 0.198 | 0.254 | -0.090 |  | 0.406 | 0.181 | -0.095 | 0.184 |
| Prev sp | -0.122 | -0.405 | -0.033 | -0.176 | 0.130 | -0.057 | 0.445 | **0.145** | 0.305 | **0.440** | **0.539** | 0.208 | **0.574** | **0.527** | 0.229 | 0.372 | 0.604 | 0.380 | 0.204 |  | **0.483** | **0.203** | 0.278 |
| Bacter-o | -0.039 | -0.399 | -0.030 | -0.209 | -0.219 | 0.065 | 0.429 | **-0.152** | **0.197** | 0.076 | **0.693** | **0.246** | **0.361** | 0.480 | -0.084 | 0.174 | **0.361** | **0.238** | 0.242 | **0.583** |  | **0.420** | **0.402** |
| SNAM | -0.173 | -0.068 | **-0.371** | -0.255 | -0.468 | -0.107 | -0.015 | **-0.071** | **0.435** | 0.167 | 0.246 | **0.252** | 0.116 | **0.084** | -0.210 | -0.177 | 0.133 | **0.416** | -0.075 | **0.212** | **0.396** |  | 0.268 |
| FUNU | -0.168 | -0.322 | -0.061 | -0.116 | -0.283 | -0.244 | **0.367** | -0.182 | 0.083 | 0.262 | 0.447 | -0.010 | **0.354** | 0.613 | -0.202 | 0.181 | 0.376 | 0.351 | -0.133 | **0.419** | **0.560** | 0.285 |  |

bolded = p<0.01
